# β‑Anomeric Mannopentaose-Based Nonenzymatic NIR Glucose Sensor Enabling Cost-Effective Continuous Monitoring

**DOI:** 10.1021/acsmeasuresciau.6c00076

**Published:** 2026-07-10

**Authors:** Sayantan Tripathy, Diana Al Husseini, Carolina I. Martinez, Anthony Dongchau, Samyucktha Ganesapandian, Melissa A Grunlan, Gerard L Cote

**Affiliations:** † Department of Biomedical Engineering, 14736 Texas A&M University, 600 Discovery Drive, College Station, Texas 77840-3006, United States; ‡ Center for Remote Health Technologies and Systems, 14736 Texas A&M University, 600 Discovery Drive, College Station, Texas 77840-3006, United States; § Department of Chemistry, Texas A&M University, 580 Ross Street, College Station, Texas 77843, United States; ∥ Department of Electrical and Computer Engineering, Texas A&M University, College Station, Texas 77840, United States

**Keywords:** concanavalin A, NIR dyes, mannopentaoses, fluorescence anisotropy, solvatochromism, implantable hydrogel

## Abstract

In this study, we present a novel, fully injectable, nonenzymatic glucose biosensing platform that uses NIR fluorescence from a PEGylated-ConA and Cy5.5-β-mannopentaose complex to achieve highly sensitive and stable detection. The proof-of-concept assay is designed for high sensitivity, long-term stability, a small size to permit injectability or minimal invasiveness, suitability across all skin tones, and cost-effectiveness. As a control, an analogous assay was prepared with the α-anomeric form (i.e., Cy5.5-α-mannopentaose), which is 10X costlier. Using fluorescence anisotropy, a computational model was developed to optimize the concentration ratios of assay components for sensitive glucose detection within the physiological range. Both conjugates demonstrated comparable responsiveness across physiological glucose concentrations, with Cy5.5-β-mannopentaose exhibiting a broader dynamic range (25–400 mg/dL) in buffer and comparable binding affinity. In addition, performance was validated in artificial interstitial fluid with Cy5.5-β-mannopentaose showing reproducible solvatochromism across the full blood glucose range (25–400 mg/dL), with strong responsiveness maintained within the physiologically relevant subcutaneous window. To further validate its ability to be housed in a membrane carrier, the Cy5.5-β-mannopentaose solution was placed in the cavity of a hollow hydrogel rod with known antifouling properties and mesh size, and >94% was retained over a 7-day period. These results establish β-mannopentaose as a promising low-cost ligand for ConA-based glucose sensing systems and support its potential use in the future development of minimally invasive and scalable glucose sensing approaches.

## Graphical abstract

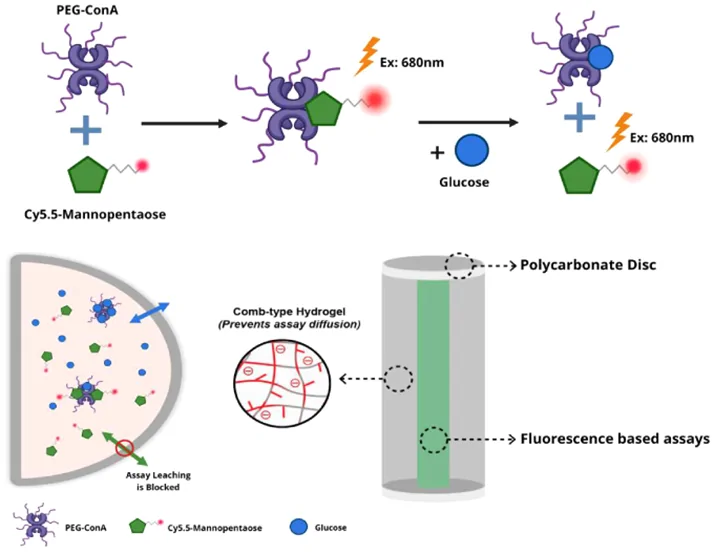

## 1 Introduction

Diabetes mellitus is a global health challenge, affecting over 500 million individuals worldwide, a number expected to rise dramatically in the coming decades.^1–3^ Self-monitoring of blood glucose via the traditional finger-prick method is limited by patient discomfort, inconvenience, and its restriction to single-point-in-time measurements.^4^ Effective management of diabetes via continuous glucose monitors (CGMs) is a promising alternative, providing real-time glucose data and enabling timely intervention to avoid complications.^5^ Over the past two decades, electrochemical indwelling CGMs have dominated the commercial landscape with major contributions from companies such as Abbott, Medtronic, and Dexcom.^6–8^ These systems offer frequent glucose measurements, typically every 1 to 15 min, and provide alerts for hypo- and hyperglycemia.^5^ These CGMs function by employing glucose oxidase, which catalyzes the oxidation of glucose to gluconolactone and hydrogen peroxide.^9^ The glucose concentration is then correlated to the hydrogen peroxide produced, often with the help of electrocatalysts. More recently, some systems have utilized the electrochemical analysis of reduced flavin adenine dinucleotide (FADH_2_) as a readout.^10^ This detection approach utilizes a direct physical connection between the subcutaneously embedded biosensing system and the external electronics adhered to the skin surface. Despite their clinical utility, enzymatic CGMs suffer from many intrinsic limitations. These include temperature and oxygen dependency, loss of enzymatic activity due to biofouling, variability in performance, and reduced stability over extended use.^11^ In addition, they require regular replacement of transcutaneous sensors every 5 to 14 days. Since they are indwelling and not fully injected, they can cause skin irritation, rashes due to prolonged adhesive use, inaccuracies if the sensor is bent or compressed (e.g., compression lows) and in some cases, sensor dislodgement.^12,13^ Moreover, many of these systems necessitate frequent calibration with fingerstick blood glucose readings to maintain accuracy, adding to user burden and reducing convenience. Despite being rare, the transcutaneous nature of these devices also poses a risk of infection.^14^ In addition, variations in hematocrit levels can influence glucose measurements in several electrochemical and optical sensing platforms by altering diffusion characteristics, signal transduction efficiency, and optical scattering properties, thereby contributing to measurement variability and reduced accuracy across patient populations.^15^ The widespread adoption of enzymatic CGMs is also hindered by economic barriers, particularly for underserved and uninsured populations.^16,17^ These systems are sometimes not fully covered by insurance, and the high recurring costs of sensors, transmitters, and calibration supplies can place a significant financial burden on patients. As a result, access to real-time glucose monitoring remains limited for many individuals who could benefit most from tight glycemic control. This economic inaccessibility further emphasizes the urgent need for affordable, long-lasting, and low-maintenance nonenzymatic sensing alternatives that can bridge the gap in diabetes care across diverse socioeconomic backgrounds.^18^ The Senseonics Eversense E3 CGM represents an advancement in nonenzymatic, optical glucose sensing, utilizing a diboronic acid-modified hydrogel with embedded electronics and optics for fluorescence-based glucose detection.^19–22^ Approved for up to 180 days of continuous use, it measures glucose via fluorescence modulation in response to glucose binding to 1,2-bis-boronate moieties.^23^ However, its reliance on UV/blue-emitting fluorophores necessitates an internal LED, photodetector, and electronics, leading to a bulky implant (3.5 mm × 18.3 mm, Ø × *d*) that requires small surgical insertion and an external skin-adhered transmitter.^24,25^ Furthermore, the total cost of use can be prohibitive without adequate insurance coverage. These design constraints, including risks of skin irritation, high cost, component failure, and the need for a surgical intervention, highlight the need for an alternative solution.

Overall, the limitations of existing CGMs have underscored the development of new glucose-monitoring systems (Table 1). As described herein, the adoption of red and near-infrared (NIR) fluorophores presents a promising direction for optical glucose sensing because of reduced light scattering and lower background autofluorescence in biological tissues. This approach may offer potential applicability across diverse skin pigmentations. Unlike UV or blue-emitting fluorophores, which require high-energy excitation and suffer from limited tissue penetration, NIR fluorophores benefit from reduced light scattering and lower background autofluorescence in biological tissues.^26^ This spectral window enables deeper signal transmission^27^ and detection through the skin, eliminating the need for embedded excitation sources such as internal LEDs as well as the associated detectors and electronics.^28^ Consequently, NIR-based biosensors can be engineered in significantly smaller form factors, allowing for subcutaneous injection, rather than surgical implantation. Besides, external excitation and detection further simplify the biosensor design, enhancing reliability. The integration of a carrier that minimizes biofouling would further promote the biosensor’s longevity. Collectively, these attributes support the development of a compact, longer-lasting, and universally useful CGM.

**Table 1:** On-the-Market and Emerging Glucose Monitoring Systems

System	Read Out	Dynamic Range (mg/dL)	Cost	Stability/sensor life	Reference
FreeStyle Libre 3 by Abbott	Amperometric electrochemical	40–400	Approx. $115/sensor	14 days	https://pro.freestyle.abbott/content/dam/adc/pro/countries/uk-en/documents/FSL3.pdf
G7 by Dexcom	Amperometric electrochemical	40–400	Approx. $100/sensor	10 days	https://res.onemed.com/DK/Produktblad/8098284-2.pdf
Dexcom G6 Pro	Amperometric electrochemical	40–400	Approx. $180/sensor	10 days	https://ctc-ri.org/sites/default/files/uploads/Dexcom G6 Pro User Guide.pdf
Eversense E3	Fluorescence-based	40–400	Approx. anually $2600/sensor	Up to 6 months	https://www.accessdata.fda.gov/cdrh_docs/pdf16/P160048S016B.pdf https://www.healthline.com/diabetesmine/eversense-e3-six-month-implantable-sensor
Guardian Connect CGM by Medtronic	Amperometric electrochemical	40–400	Approx. $60/sensor	7 days	https://type1better.com/wp-content/uploads/2022/12/CGM-comparison-table.pdf
Nanoenzyme composite based biosensor	Amperometric	0.02–144	Not reported	45 days	^29^
Gel electrolyte graphene transistor-based biosensor	Current response	0.0002–450	Not reported	15 days	^30^
Flower-like CoO nanowire-decorated Ni foam-based biosensor	Amperometric (Saliva)	0.09–45.5	Not reported	21 days	^31^
Hollow Ni-Al-Mn triple-layered hydroxide (HLTH) nanocomposite-based biosensor	Amperometric (Saliva)	0.27–144	Not reported	30 days	^32^
Implantable silicon-on-insulator (SOI) photonic chip	NIR absorption spectroscopy	18–1261	Not reported	Not reported	^33^
Chip-based near-infrared (NIR) sensor	Transmission NIR spectroscopy combined with microdialysis	60–350	Not reported	Not reported	^34^
**This sensing approach**	**NIR-Fluorescence**	**25–400**	*Leverages lower cost		

Among strategies using NIR fluorescent dyes, mannose-based sensing has gained attention, particularly in systems using Concanavalin A (ConA), a protein that binds to glucose and mannose.^35^ When incorporated into a competitive binding assay with a mannose-containing ligand, ConA can be used to reversibly and selectively bind to glucose without requiring enzymatic reactions. However, native ConA has solubility and aggregation issues that limit its practical application.^36^ PEGylated-ConA offers improved solubility, reduced nonspecific interactions, and greater stability for biosensing applications.^37,38^ However, in ConA-based assays, the choice of the mannose ligand with NIR dye is crucial for optimizing sensitivity and response range. While earlier studies have used mannobiose, mannotriose, and mannotetraose, these shorter oligomers offer limited binding valency in implantable hydrogels and are also expensive.^20,39^ We hypothesized that mannopentaose (i.e., five mannose units) could provide enhanced multivalency, potentially increasing the strength and stability of binding with PEGylated-ConA and improving glucose displacement dynamics as indicated by some previously reported proof-of-concept studies with mannooligosaccharides.^39^ Interestingly, previous studies have described the binding superiority of α-anomeric forms of mannooligosaccharides,^40^ largely due to their favorable conformations in molecular docking and reported higher binding scores with the protein. However, the potential of the β-anomer, particularly in the context of multivalent ligands like mannopentaose, remains largely unexplored. This oversight is significant given that β-anomeric mannooligosaccharides are relatively 8–10X less costly and may also offer comparable performance in competitive binding assays due to distinct hydrogen bonding profiles and steric characteristics.^41^

In this study, we systematically evaluate β-mannopentaose as a ligand in a ConA-based competitive glucose sensing system and compare its performance with the corresponding α-anomer. Motivated by its substantially lower cost (approximately 8–10X reduction), we investigate whether the β-anomer can sustain comparable binding affinity and glucose-responsive behavior. Cy5.5-labeled α- and β-mannopentaose conjugates were synthesized and characterized using fluorescence anisotropy and solvatochromic response measurements in buffer and artificial interstitial fluid. In addition, hydrogel encapsulation was examined to assess material compatibility relevant to potential implantable sensing configurations. An α-mannopentaose analogue was likewise prepared. We characterized the polarity-sensitive response of these conjugates, observing distinct fluorescence changes upon complexation due to the solution-environmental sensitivity of the Cy5.5. To further validate and quantify glucose responsiveness, fluorescence anisotropy experiments were conducted which showed sensitive and reproducible detection of both hypo- and hyperglycemic conditions. To evaluate translational potential, we further tested the PEGylated-ConA−β-mannopentaose–Cy5.5 platform in artificial interstitial fluid (ISF), where glucose levels represent ∼45–78% of blood glucose. The assay retained robust solvatochromic and anisotropy responses across 25–400 mg/dL, fully covering the physiologically relevant subcutaneous window spanning hypoglycemic, normoglycemic, and hyperglycemic conditions. The developed assay was injected into the hollow cavity of a known mechanically robust antibiofouling hydrogel membrane, and retention of the assay was determined to evaluate the efficacy of the design. Overall, this work establishes a cost–performance optimization framework for ConA-based competitive binding assays and provides design insights for the development of compact optical glucose sensing systems.

## 2 Materials and Methods

### 2.1 Materials

Tris-buffered saline tablets (Sigma), sodium carbonate, 1-(*N*-Boc-aminomethyl)-4-(aminomethyl)­benzene, sodium bicarbonate, *N*-isopropylacrylamide (NIPAAm, 97%), 2-acrylamido-2-methylpropane sulfonic acid (AMPS, 97%), *N*-vinylpyrrolidone (NVP, ≥ 99%), *N*,*N*′-methylenebisacrylamide (BIS, 99%), 2-hydroxy-4′-(2-hydroxyethoxy)-2-methylpropiophenone (Irgacure 2959, 98%), dimethyl sulfoxide (DMSO), acetic acid, sodium cyanoborohydride (NaBH_3_CN), trifluoroacetic acid (TFA), methoxypolyethylene glycol 5000 propionic acid *N*-succinimidyl ester, tri-isopropyl silane (TIPS), diethyl ether, *N*,*N*-diisopropylethylamine (DIPEA), dimethylformamide (DMF), ConA Type IV lyophilized powder, methyl α-d-mannopyranoside, and Amicon Ultra-2 Centrifugal Filters Unit 30 kDa were purchased from Sigma (St. Louis, MO). Cyanine5.5 (Cy5.5) NHS ester was purchased from Lumiprobe (Hunt Valley, Maryland). The sugars α1–3,α1–6 mannopentaose and 1,4-β-D-mannopentaoses were purchased from Neogen. The Tris buffer (pH 7.4) and 0.1 M carbonate-bicarbonate buffer (pH 8.5) were prepared in nuclease-free water.

### 2.2 Computational Binding Model

To guide assay optimization and predict glucose-responsive signal transduction, a computational model was implemented that simulated the competitive binding dynamics between PEGylated-ConA, glucose, and Cy5.5-α-mannopentaose or Cy5.5-β-mannopentaose. These model equations were adapted from our previously established competitive binding framework and were solved analytically to determine optimal concentration ratios of assay components for maximal sensitivity within the physiological glucose range.^20^ In summary, the model assumes a reversible competitive binding mechanism governed by the following equilibria:

1 C+M⇌CM,C+G⇌CG

where *C* is free ConA, *M* is free mannopentaose-conjugate, and *G* is free glucose. The bound complexes *CM* and *CG* represent ConA bound to mannopentaose and glucose, respectively. The dissociation constants *K*_M_ and *K*_G_ are defined by:

2 $$K_{M}=\frac{\left[C\right]\left[M\right]}{\left[\mathrm{CM}\right]},,K_{G}=\frac{\left[C\right]\left[G\right]}{\left[\mathrm{CG}\right]}$$

Mass conservation yields the following constraints:

3 $${[M]}_{0}=\left[M\right]+\left[\mathrm{CM}\right]$$

4 $${[G]}_{0}=\left[G\right]+\left[\mathrm{CG}\right]$$

5 $${[C]}_{0}=\left[C\right]+\left[\mathrm{CM}\right]+\left[\mathrm{CG}\right]$$

Substituting and rearranging yield explicit expressions for [CM] and [CG]:

6 $$\left[\mathrm{CM}\right]=\frac{\left[C\right]{[M]}_{0}}{K_{M}+\left[C\right]}$$

7 $$\left[\mathrm{CG}\right]=\frac{\left[C\right]{[G]}_{0}}{K_{G}+\left[C\right]}$$

Substituting into the ConA conservation equation produces a cubic equation in [C]:

8 $${[C]}^{3}+x{[C]}^{2}+y\left[C\right]+z=0$$

with the coefficients:

$$\begin{array}{l} x=K_{G}+K_{M}+{[G]}_{0}+{[M]}_{0}-{[C]}_{0}\\ y=K_{M}\left({[G]}_{0}-{[C]}_{0}\right)+K_{G}\left({[M]}_{0}-{[C]}_{0}\right)+K_{G}K_{M}\\ z=-K_{G}K_{M}{[C]}_{0} \end{array}$$

The physically meaningful root (real, non-negative) was solved analytically using the trigonometric method derived by Wang.^42^ This approach is applicable when the cubic equation admits three real roots, which occurs when the discriminant of the depressed cubic is negative. In the present formulation, this corresponds to the condition:

$$x^{2}-3y>0$$

Under this condition, the solution for the free ConA concentration [C] can be written as:

9 $$\left[C\right]=-\frac{x}{3}+\frac{2}{3}\sqrt{x^{2}-3y}\;\cos\theta$$

where

10 $$\theta =\frac{1}{3}\arccos\left(\frac{-2x^{3}+9xy-27z}{2{\left(x^{2}-3y\right)}^{3/2}}\right)$$

The argument of the arccosine function is constrained to the interval [−1,1], ensuring that the solution remains real.

Among the mathematically valid roots, the physically meaningful solution was selected as the real, non-negative root that does not exceed the total ConA concentration [C]_0_.

For all parameter combinations used in this study, including *K*_G_ = 2.5 × 10^–3^ M, *K*_αM_ = 0.1 × 10^–6^ M, and *K*_βM_ = 0.07 × 10^–6^ M, this condition is satisfied, and the solution yields a unique physically admissible root.

Once [C] was computed, the unbound mannopentaose concentration was calculated via

11 $$\left[M\right]=\frac{K_{M}{[M]}_{0}}{K_{M}+\left[C\right]}$$

For this study, experimentally determined dissociation constants were used: *K*_G_ = 2.5 × 10^–3^ M^20,43^ , *K*_αM_ = 0.1 × 10^–6^ M, and *K*_βM_ = 0.07 × 10^–6^ M. Simulations were done over a matrix of initial concentrations [M]_0_ and [C]_0_ evaluating changes in the free mannose signal between the extremes of physiological glucose concentration ([G]_0_ = 0 and 400mg/dL).

The model output was quantified as the percent change in free mannose signal due to glucose:

12 $$\Delta \%=100\times \left(\frac{{[M]}_{400}-{[M]}_{0}}{{[M]}_{0}}\right)$$

This percent change was used to identify concentration regimes yielding maximal assay responsiveness to physiological glucose. Sensitivity maps generated from this model enabled rational selection of candidate concentrations for *in vitro* validation with both Cy5.5-α-mannopentaose and Cy5.5-β-mannopentaose. These simulations demonstrated high predictive utility, as experimentally validated fluorescence anisotropy shifts aligned with the regions of maximum predicted sensitivity.

### 2.3 Fabrication of Hollow Hydrogel Rods

Double network (DN) comb hydrogel hollow rods (2.5 mm × 5 mm, Ø × *l*; postequilibration) were fabricated following a modified 2-step UV cure process.^44^ An aqueous single network (SN) precursor solution comprised of DI water, NIPAAm (85 mol %), AMPS (11.25 mol %), and PAMPS methacrylated (PAMPS_10_-MA) comb macromer (3.75 mol %) was prepared at a 1.11 M total monomer concentration in a round-bottom flask (rbf). Following the complete dissolution, the rbf was wrapped in foil to avoid premature exposure to light. BIS cross-linker (3.3 mol % wrt total monomer concentration) and Irgacure 2959 (4.6 mol% wrt total monomer concentration) were added to the rbf and stirred until dissolved. The DN aqueous precursor solution was comprised of NIPAAm (2.5 M), NVP (5% w/v), BIS (0.15 mol % wrt NIPAAm), and Irgacure 2959 (2 mol % wrt NIPAAm). Reagents were added to a secondary rbf in a similar manner as to those described in the SN solution.

The SN solution was injected into a cylindrical glass mold prepared with the following dimensions: ∼6 mm × ∼1 mm × ∼20 mm, Ø × *ID* × *l*). Parafilm was used to seal the ends of the mold. Custom-made Teflon-coated caps with a hole (∼400 μm) were placed on the ends. A 400 μm stainless-steel wire was inserted through the cap, creating the hollow cavity. The assembled mold and cap were placed into an ice–water bath to ensure NIPAAm remained soluble in the solution during curing and were subsequently exposed for 30 min to UV-light at 365 nm (UV-transilluminator, 6 mW/cm^2^). During the 30-min cure, the mold was rotated at 180° after 15 min. The Teflon-coated caps were subsequently removed from the mold. The hydrogel and mold construct were placed into an oven (10 min at 120 °C), causing detachment of the hydrogel from the glass mold. The resulting SN hollow hydrogel rod was submerged in the DN precursor solution for 2 days at RT. Next, a 500 μm stainless-steel wire was inserted into the central cavity of the hollow hydrogel, tightly wrapped with cling film to ensure a flush outer surface, and exposed to UV-light for 10 min, rotating 180° after 5 min. The steel wire was removed, and the resulting DN hollow hydrogel rod was placed into DI water for 3 days with daily DI water exchange. Final hydrogel rods were cut to length with a razor blade for final dimensions of ∼2.5 mm × ∼ 1 mm × ∼ 10 mm, Ø × *ID* × *l*.

The full experimental details are described in Supporting Information.

## 3 Results and Discussion

### 3.1 Principles of the Assay and Biosensor Design

Our glucose-sensing assay employs PEGylated-ConA, a multivalent carbohydrate binding protein with strong specificity for cis-diol motifs in glucose and mannose. PEGylation enhances the solubility, stability, and biocompatibility of ConA, reducing aggregation under physiological conditions which enables reliable integration into the sensor platform.^38^ In this system, Cy5.5-labeled mannopentaose (α and β anomers) binds with high affinity to the carbohydrate recognition domains of PEGylated-ConA, producing a stable NIR fluorescence signal (Figure 1A). Cy5.5 was chosen for its polarity sensitivity, deep tissue penetration, and minimal autofluorescence, which enhance signal fidelity in subcutaneous environments.^45^ Previous studies using Cy5.5-labeled mannose-based ligands have also demonstrated consistent responses of this fluorophore under different skin pigmentation roughly analogous to human Fitzpatrick skin types I–II, III–IV, and V–VI, which supports its suitability for subcutaneous applications.^46^ Upon glucose introduction, competitive binding displaces the Cy5.5-labeled mannopentaose from the PEGylated-ConA (Figure 1B). This displacement shifts the fluorophore from a hydrophobic, rigid environment to a more polar aqueous phase, resulting in a proportional decrease in fluorescence intensity. This solvatochromism shift, previously well documented for Cy5.5-labeled glycoconjugates, enables a quantitative fluorescence response that correlates with glucose concentration.^46^ The use of fluorescence solvatochromism is valuable for assay characterization, but the final biosensor system would rely on a fluorescence modulation transduction mechanism (e.g., Fluorescence Resonance Energy Transfer or fluorescence quenching) rather than enzymatic catalysis, offering a reversible, real-time sensing mode with reduced susceptibility to enzymatic degradation or cofactor depletion. Complementary fluorescence anisotropy assays further confirm glucose-induced displacement by detecting changes in the rotational mobility of the Cy5.5-ligand. Together, these mechanisms enable reversible, real-time sensing with high specificity and robustness for subcutaneous glucose monitoring.

**Figure 1:**
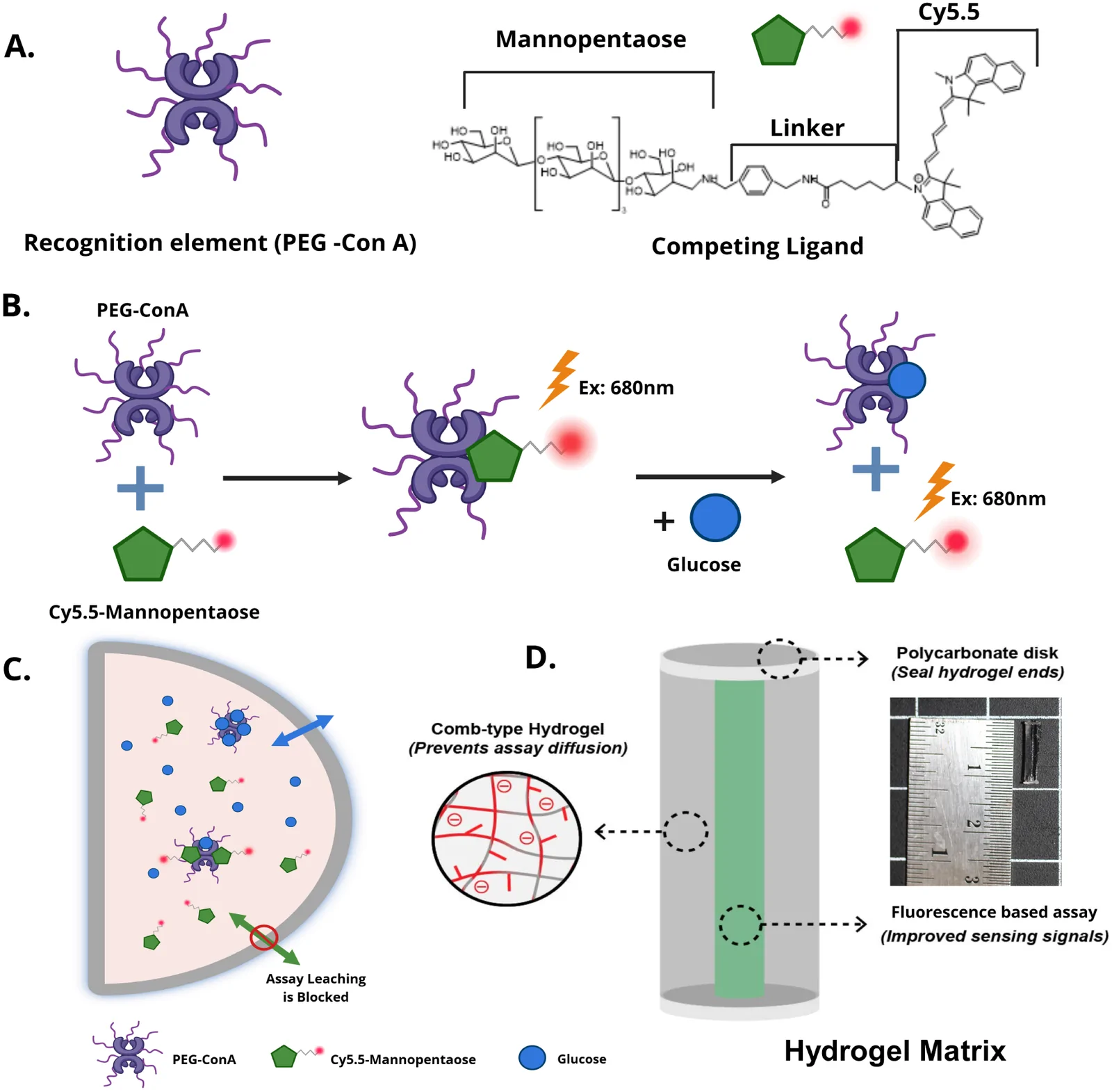
(A) The recognition element, PEGylated-ConA, binds to a Cy5.5-labeled mannopentaose (depicted here for the α-anomer) via a competitive ligand interaction. (B) In the absence of glucose, Cy5.5-labeled mannopentaose binds to PEGylated-ConA, producing fluorescence upon excitation at 680 nm. Upon glucose addition, displacement of the labeled ligand reduces fluorescence solvatochromism, enabling glucose quantification for this characterization. (C) The sensing assay solution is encapsulated within the hollow cavity of a DN comb-type hydrogel whose mesh size limits assay molecule leaching. (D) The complete biosensor implant design includes sealing the hydrogel cavity with polycarbonate discs.

A thermoresponsive DN comb hydrogel rod was used as a potential membrane to house the developed assay (Figure 1C). This hydrogel carrier was selected due to its expected antibiofouling behavior, advantageous mechanical robustness, and size-selective diffusion properties. Owing to its low mesh size (1 nm < ξ < 3 nm), the hydrogel is capable of retaining small molecules within the cavity, while permitting the diffusion of glucose (*D*_h_ ∼ 1 nm) (Dong et al., 2020). Thus, successful retention of the assay was expected. The compressive modulus (*E*) and strength (σ_f_), ∼0.6 MPa and ∼1 MPa, respectively (Dong et al., 2020), would ensure successful implantation and subsequent indwelling within the subcutaneous tissue. Additionally, mitigation of biofouling is anticipated based on its high hydration (∼90%) and thermosensitivity, as demonstrated for similar hydrogels in our prior report (Means et al., 2019). The assembled biosensor, with the ends sealed with polycarbonate caps, provides a convenient small, cylindrical geometry (∼2.5 mm × ∼11 mm, Ø × l), suitable for subcutaneous injection (Figure 1D). This design supports injectable, point-of-care applications allowing the sensor to maintain functionality under dynamic physiological conditions.

### 3.2 Molecular Docking Study of Mannose Sugars with ConA

To gain preliminary structural insight into the potential interaction between mannopentaose and ConA prior to experimental evaluation, molecular docking simulations were performed. As computational modeling has been widely used as a supporting tool in glycan–protein interaction studies, these simulations were employed here to guide experimental design and visualize possible binding orientations rather than to derive quantitative binding energetics.^47^ The SMILES structures of conventional α-anomeric mannose oligosaccharides (mannobiose, mannotriose, and mannotetraose), along with both α- and β-mannopentaose (Figure 2), were analyzed to examine their possible binding modes within the ConA carbohydrate recognition domain. The docking poses indicate that all mannopentaose derivatives can occupy the binding pocket and form multiple potential hydrogen-bonding interactions with key amino acid residues. Compared to shorter oligosaccharides such as mannotetraose, mannopentaose exhibited a greater number of potential hydrogen-bonding interactions (Table S1), consistent with its extended glycan structure and increased availability of hydroxyl groups for interaction. These additional contact points are consistent with the multivalent nature of the ligand and align with previously reported roles of multivalency in glycan–lectin interactions.^48^ Both α- and β-mannopentaose exhibited comparable docking scores (−5.4 and −5.0, respectively) and adopted similar binding poses within the ConA carbohydrate recognition domain. Both anomers formed multiple contacts with key amino acid residues, indicating similar binding site engagement patterns. Previous studies on ConA–ligand systems have suggested that hydrogen-bond interactions may contribute to ligand–protein association; however, such effects cannot be quantitatively assessed from the present docking analysis.^49^ Importantly, docking results should be interpreted strictly as qualitative representations of possible ligand binding poses. Due to the rigid receptor approximation, absence of explicit solvent, lack of full energy minimization, and limited conformational sampling, these results are not intended to provide quantitative binding free energies or to distinguish relative binding affinities between ligands. To further assess steric compatibility with the hydrogel matrix, the approximate molecular lengths of mannopentaose and mannotetraose were estimated computationally using Avogadro software. We calculated the probable molecular length of α-mannopentaose (approximately **47.87 Å**, Figure S5) compared to α-mannotetraose (approximately 38.84 Å, Figure S6) using Avogadro software. The longer mannopentaose structure suggests improved steric accommodation within the polymer matrix, which may contribute to reduced leaching and more sustained encapsulation in the implantable biosensor. Overall, docking provides structural context supporting the selection of mannopentaose as a ligand for experimental evaluation in the competitive glucose-sensing platform.

**Figure 2:**
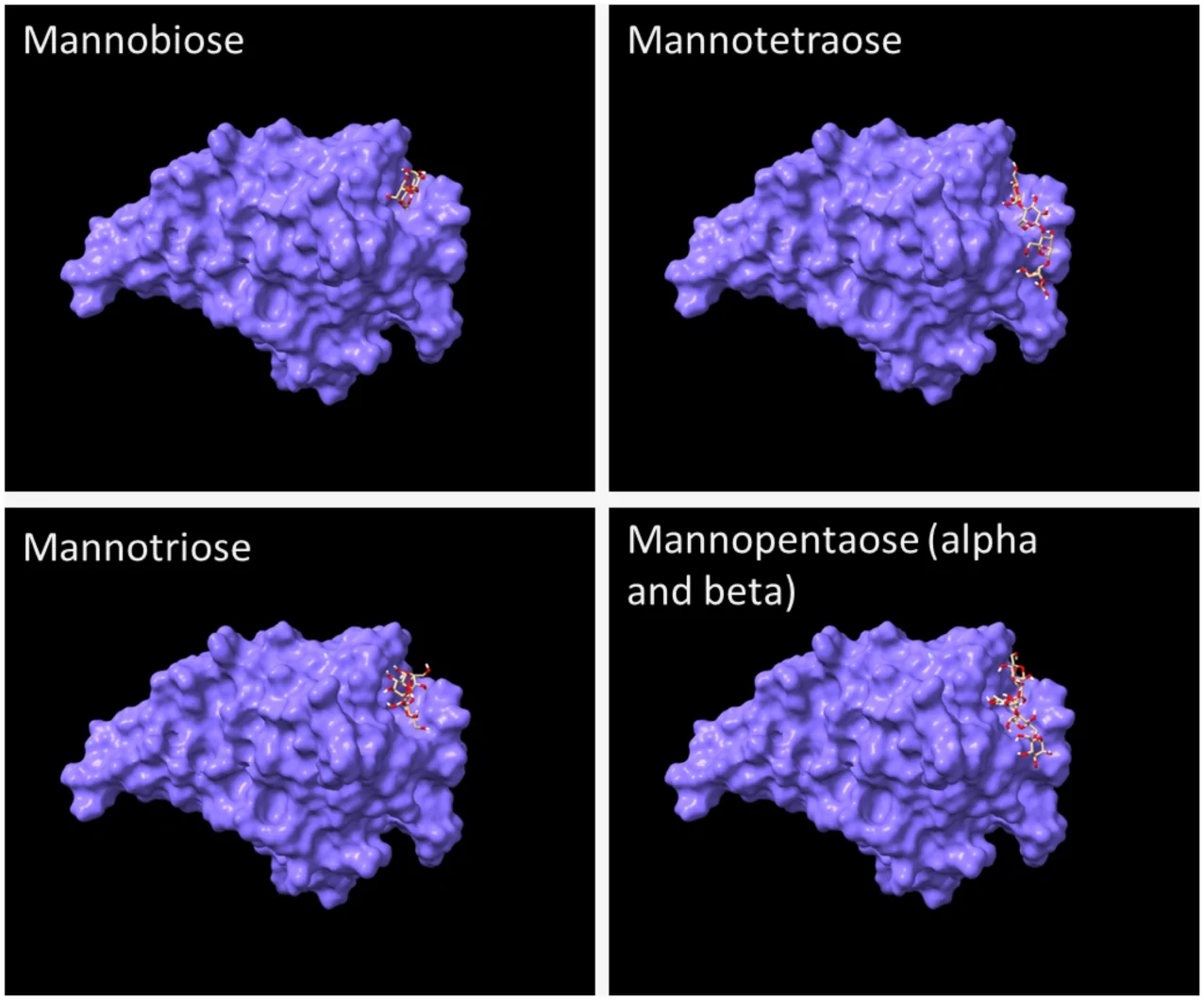
Surface representations showing docking of various mannose-containing oligosaccharides including mannobiose, mannotriose, mannotetraose, and mannopentaose (α and β anomers) within the ConA binding site. Binding poses illustrate increasing molecular interaction as saccharide length increases.

### 3.3 Characterization of Cy5.5-Labeled Mannopentaoses

Following the confirmation of mannopentaose (both anomers) binding capability with ConA through docking studies, we adapted a synthetic strategy to prepare fluorescently labeled ligands suitable for the competitive glucose assay (Figure S1). A small molecule linker, 1-(*N*-Boc-aminomethyl)-4-(aminomethyl)­benzene, was used to attach the mannose ligands to the Cy5.5 fluorescent dye. A compact design helps the construct diffuse more rapidly, which can improve binding efficiency when the interaction surface of the ligand remains constant. The synthesis involved reductive amination to attach either α- or β-mannopentaose to the linker via its free amine group. A second amine group on the linker was initially protected by a BOC group to prevent unwanted reactions with a second sugar molecule. This protection was essential to ensure high product yield by avoiding symmetrical or double-ended glycan conjugation.^20^ After deprotecting the BOC group, a Cy5.5 NHS ester was introduced to label the molecule with Cy5.5. The final products were verified by mass spectrometry and confirmed successful conjugation as well as purity. For the α-anomer, the dominant ion charges appeared at *m*/*z* 756.85–759.86 (Figure S2), matching the simulated isotope distribution at the charged state yielded the expected mass of 1515 Da confirming the correct molecular composition (C_78_H_106_N_4_O_26_) and successful synthesis. In contrast, the β-anomer spectrum displayed the singly charged ion series at *m*/*z* 755.84–759.86 (Figure S3) is also consistent with the calculated exact mass of 1515 Da confirming the correct molecular composition (C_78_H_106_N_4_O_26_). In both cases, the measured isotope patterns aligned closely with theoretical predictions, confirming successful Cy5.5 conjugation without detectable mass deviation. Fluorescence measurements further confirmed that the Cy5.5-labeled mannopentaoses retained the expected optical properties of Cy5.5. This method provides a reliable and efficient route for synthesizing fluorescently labeled α- and β-mannopentaose. To support glycan-binding assays and prevent protein aggregation, PEGylated-ConA was also prepared with the previously reported protocol.^38^ In dynamic light scattering (DLS) experiment, PEGylated-ConA had a majority uniform particle size distribution of around 32 nm and minimal aggregation, as indicated by a lower PDI of 0.221 (Figure S4). Overall, this approach is straightforward, reproducible, and adaptable to other glycans or dyes.

### 3.4 Determination of Optimal Pegylated-ConA Concentration with Cy5.5-Labeled Mannopentaoses for the Polarity-Based Fluorescent Solvatochromism Assay

After confirming the mannopentaoses’ binding relative to shorter mannooligosaccharides, glucose-sensing performance was evaluated using the fluorescence solvatochromism assay, with protein–ligand complex formation and polarity-based solvatochromism shown in Figure 3A and B. To first confirm that Cy5.5 retains its optical properties following conjugation, UV–Vis absorbance and fluorescence emission spectra of free Cy5.5 and Cy5.5–mannopentaose (α and β) were recorded (Figure S7A,B). The characteristic near-infrared absorption and emission peaks of Cy5.5 were preserved after conjugation, with only minor spectral variations observed, confirming that fluorophore functionality is maintained within the sensing construct. To evaluate whether Cy5.5 exhibits nonspecific interactions with ConA, we compared four conditions: (i) free Cy5.5 (0.1 μM) with ConA (0.4 μM), (ii) free Cy5.5 (0.1 μM) with PEGylated-ConA (0.4 μM), (iii) β-mannopentaose–Cy5.5 (0.1 μM) with PEGylated-ConA (0.4 μM), and (iv) free Cy5.5 (0.1 μM) in buffer. After incubation, samples were passed through 30 kDa centrifugal filters to remove unbound dye, and fluorescence was measured in the retentates (Figure S7C). Both free Cy5.5 incubated with ConA and free Cy5.5 incubated with PEGylated-ConA showed only minimal fluorescence, whereas β-mannopentaose–Cy5.5 with PEGylated-ConA produced a markedly stronger signal. This demonstrates that fluorescence retention occurs only when the β-mannopentaose moiety mediates specific binding to the ConA carbohydrate recognition domain. A small residual signal was observed in the control samples lacking β-mannopentaose. However, this was comparable to the minimal fluorescence also detected when free Cy5.5 alone was incubated in buffer and filtered, indicating that the effect originates from partial retention of dye aggregates by the 30 kDa membrane rather than protein-mediated interactions. Taken together, these results confirm that the fluorescence enhancement arises from specific β-mannopentaose–ConA binding, which localizes Cy5.5 within a less polar microenvironment and increases emission through solvatochromism. Following that, the fluorescence response of both 0.4 μM PEGylated-ConA coupled Cy5.5−α-mannopentaose (Figure S7D) and 0.4 μM PEGylated-ConA coupled Cy5.5−β-mannopentaose systems (Figure S7E) was evaluated using full emission spectra recorded in the absence and presence of glucose (50 mg/dL). In both cases, glucose addition resulted in a reproducible decrease in emission intensity at the Cy5.5 emission maximum without any observable shift in the emission peak.

**Figure 3:**
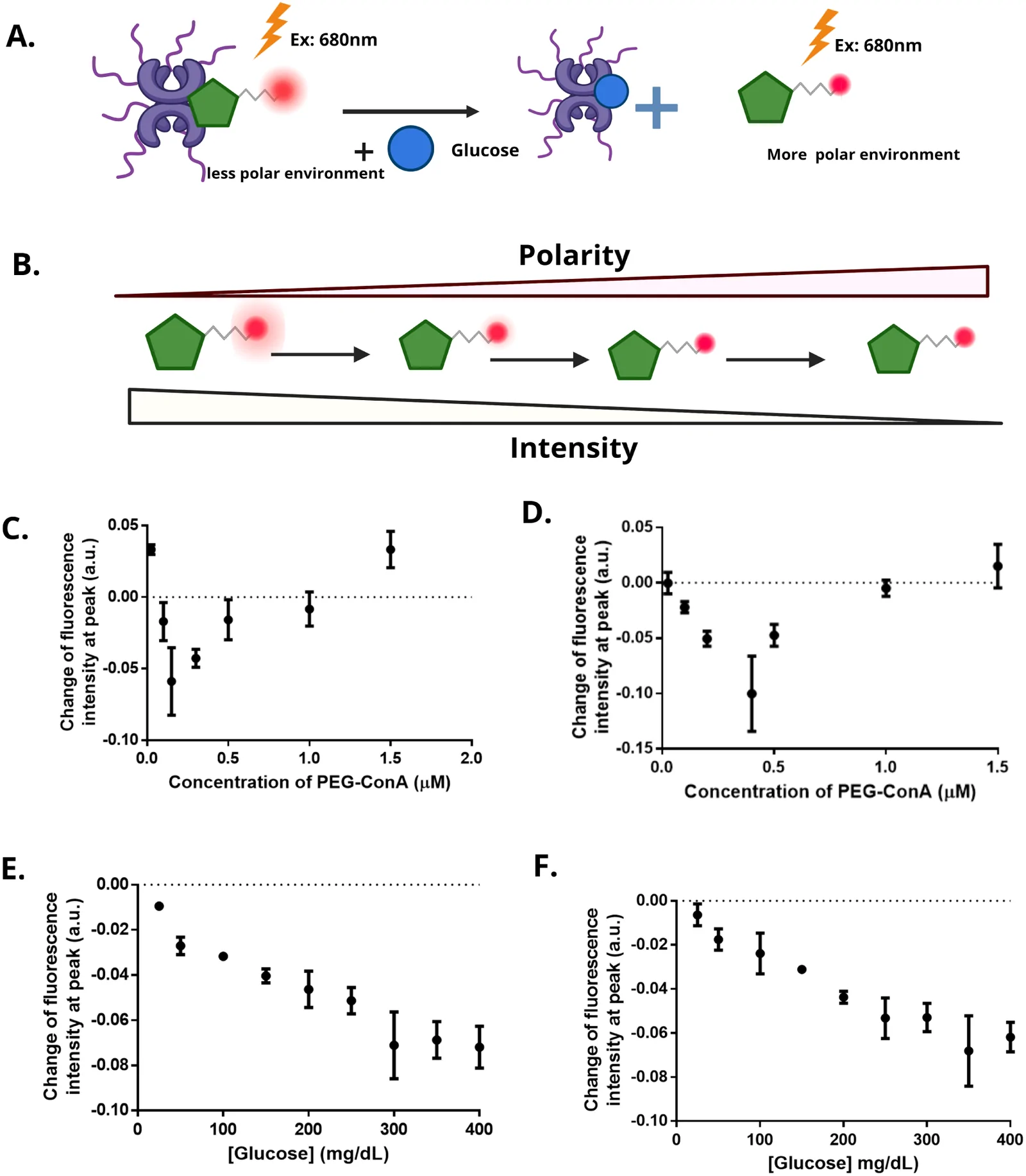
Competitive fluorescence-based glucose sensing using PEGylated-ConA and Cy5.5-labeled mannopentaose. (A) Schematic of the assay: PEGylated-ConA binds Cy5.5-mannopentaose, giving high fluorescence (excitation 680 nm). Glucose competitively displaces the conjugate, reducing signal. (B) Binding places Cy5.5 in a low-polarity environment, enhancing fluorescence; glucose displaces the conjugate, shifting Cy5.5 to aqueous surroundings and decreasing emission via solvatochromism. (C–D) Optimization of PEGylated-ConA concentration for complex stability: (C) 0.15 μM for Cy5.5-α-mannopentaose; (D) 0.4 μM for Cy5.5-β-mannopentaose. (E–F) Glucose response curves (25–400 mg/dL): (E) Cy5.5−α-mannopentaose (0.1 μM) with PEGylated-ConA (0.15 μM) exhibited a near-linear response up to ∼300 mg/dL followed by a signal plateau; (F) Cy5.5−β-mannopentaose (0.1 μM) with PEGylated-ConA (0.4 μM) displayed a broader dynamic range across 25–400 mg/dL. Error bars represent standard deviation (*n* = 3).

To determine the optimal receptor-to-ligand ratio, PEGylated-ConA was first titrated against a fixed concentration (0.1 μM) of Cy5.5-α- or β-mannopentaose (0.025, 0.1, 0.2, 0.4, 0.5, 1, 1.5 μM) in the presence and absence of glucose. For α-mannopentaose, the change in fluorescence intensity increased with PEGylated-ConA concentration and plateaued at 0.15 μM (Figure 3C), indicating near-complete complex formation. In contrast, β-mannopentaose required ∼0.4 μM PEGylated-ConA to reach maximal solvatochromic modulation. Because anisotropy measurements indicated comparable binding affinity between anomers, this higher nominal receptor requirement is interpreted as an anomer-dependent difference in fluorescence transduction efficiency rather than weaker equilibrium binding. Despite requiring a higher receptor concentration, β-mannopentaose forms a stable complex and, being nearly 10× less expensive than the α-anomer, represents a cost-effective alternative for scalable or disposable glucose sensor applications. After confirming the required concentration of PEGylated-ConA for stable complex formation, competitive displacement assays were performed to evaluate glucose responsiveness across physiologically relevant concentrations (25–400 mg/dL). Both α- and β-mannopentaose complexes exhibited a dose-dependent decrease in fluorescence upon glucose addition, with near-linear behavior (25–300 mg/dL) before saturation (Figure 3E,F). Overall, these results establish the conditions for maximal fluorescence modulation and confirm that both α- and β-mannopentaose form stable, responsive complexes suitable for quantitative glucose sensing. Although variability was higher at elevated glucose concentrations (Figure 3E,F), this is attributed to the system approaching saturation in the competitive displacement regime, where small variations in independent sample preparation resulted in magnified changes in fluorescence response. To independently assess the contribution of photobleaching under the applied measurement conditions, a control experiment was performed using PEGylated-ConA and Cy5.5-β-mannopentaose in the absence of glucose. The sample was subjected to six consecutive fluorescence measurements under identical excitation conditions over a 6-min period, showing only ∼1.5% decrease in signal (Figure S7F), indicating negligible photobleaching under the experimental conditions. Using these optimized conditions, the binding stoichiometry and dynamic behavior of the assay under competitive conditions were further characterized with fluorescence anisotropy.

### 3.5 Binding Study with Cy5.5-Labeled Mannopentaoses, and Evaluation of Computational Model

To validate the binding interactions observed in the previous solvatochromism assays, fluorescence anisotropy measurements were performed between PEGylated-ConA and Cy5.5-labeled mannopentaoses. Rotation of the Cy5.5-mannopentaose ligand upon binding to the larger PEGylated-ConA complex is restricted, resulting in increased anisotropy. Upon addition of glucose, displaced and free Cy5.5-mannopentaose exhibit lower anisotropy due to rapid rotational diffusion (Figure 4A), providing a direct readout of specific mannopentaose–PEGylated-ConA interactions. Titration of PEGylated-ConA with Cy5.5-α-mannopentaose revealed strong binding, with an apparent dissociation constant (*K*_d_) of 0.10 μM (Figure 4B). Cy5.5-β-mannopentaose exhibited a comparable interaction, with an apparent *K*_d_ of 0.07 μM (Figure 4C). The fitted one-site binding curves and corresponding 95% confidence intervals are shown in Figure 4B and C. These results indicate that both anomers form stable complexes with PEGylated-ConA, with the β-anomer showing a slightly lower fitted *K*_d_ at the point estimate level. However, the 95% confidence intervals overlap substantially (see Section S1.7), indicating that the differences between the two anomers are comparable. Residual analysis metrics showed (Figure S8A­(i and ii)) no evidence of systematic deviation from the one-site binding model. This anisotropy-based approach further supports the viability of mannopentaose derivatives in the nonenzymatic glucose-sensing platform.

**Figure 4:**
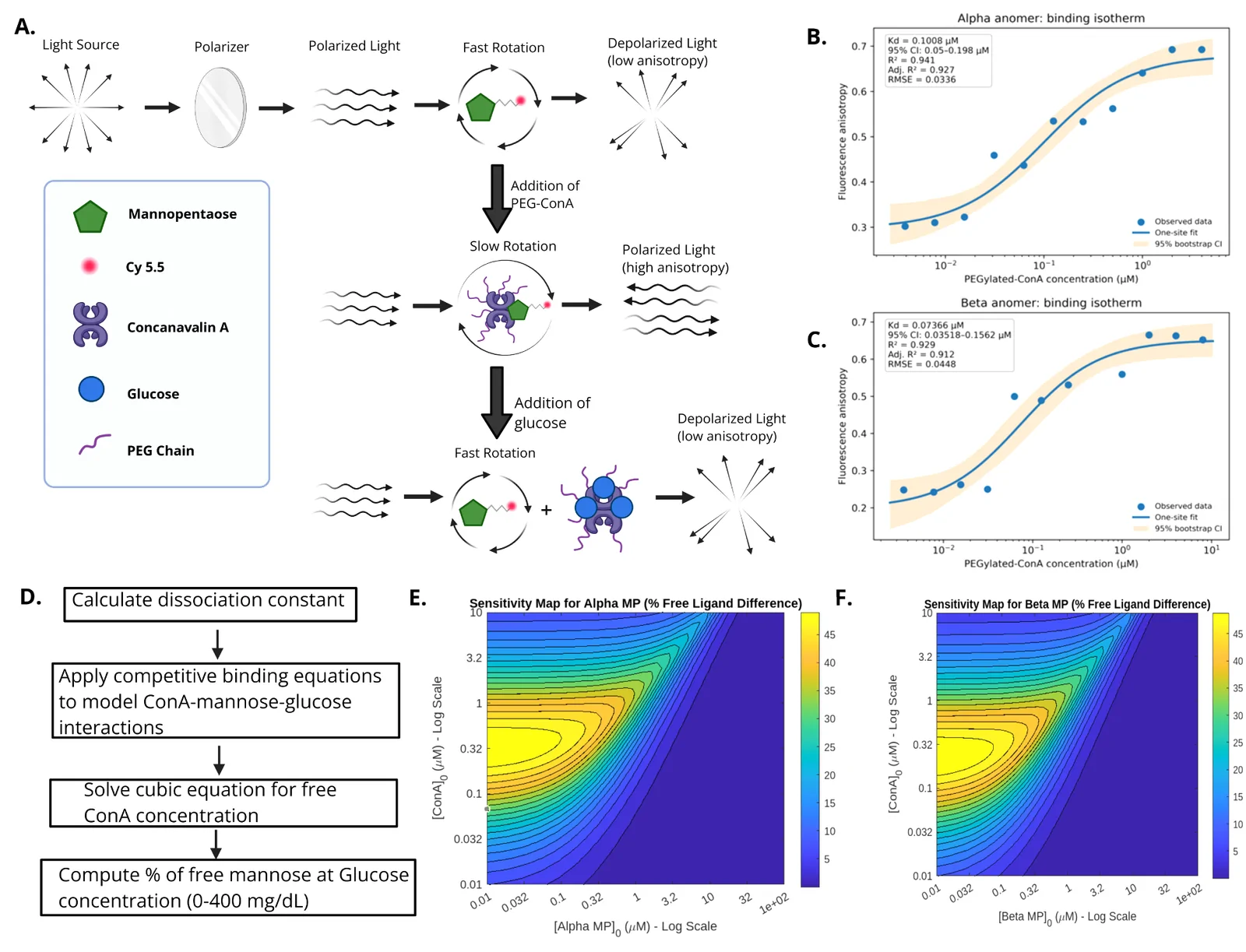
Fluorescence anisotropy-based binding of Cy5.5-labeled mannopentaose to PEGylated-ConA. (A) Schematic of the assay: free Cy5.5-mannopentaose rotates rapidly (low anisotropy), whereas binding to PEGylated-ConA slows rotation (high anisotropy). Glucose competes for binding, releasing the labeled ligand and restoring low anisotropy. (B) Binding isotherm of PEGylated-ConA with Cy5.5-α-mannopentaose, showing the one-site binding fit and 95% confidence interval. (C) Binding isotherm of PEGylated-ConA with Cy5.5-β-mannopentaose, showing the one-site binding fit and 95% confidence interval. (D) Computational workflow: anisotropy-derived *K*_d_ values were used to model competitive binding of glucose and mannopentaose, calculating free PEGylated-ConA and ligand levels across glucose concentrations (0–400 mg/dL). (E–F) Sensitivity maps for (E) α- and (F) β-mannopentaose show conditions with the highest glucose detection sensitivity (yellow).

To guide the experimental optimization of glucose sensitivity performance, a computational competitive binding model was constructed using the *K*_d_ values of Cy5.5-α-mannopentaose (1.0 × 10^–7^ M) and Cy5.5-β-mannopentaose (7.0 × 10^–8^ M), the known glucose–ConA dissociation constant (2.5 × 10^–3^ M), and the physiological glucose range (0–400 mg/dL) as input parameters (Figure 4D). The model aimed to identify optimal concentrations of ligand and receptor that maximize the percent change in free mannopentaose upon glucose addition. For Cy5.5-α-mannopentaose, the sensitivity map predicted an optimal PEGylated-ConA concentration of 0.32 μM at 0.1 μM ligand, with the region between 0.1–0.6 μM showing free ligand changes exceeding 35%, and the highest predicted sensitivity above 40% (Figure 4E). For Cy5.5-β-mannopentaose, optimal PEGylated-ConA concentrations were predicted around 0.2–0.4 μM, with a broader plateau of high sensitivity (Figure 4F). The computational model identified concentration regimes expected to provide high glucose sensitivity. For α-mannopentaose, the model predicted a broad high-sensitivity region spanning approximately 0.1–0.6 μM PEGylated-ConA, within which the experimentally selected concentration of 0.15 μM produced near-maximal fluorescence modulation while minimizing receptor usage. For β-mannopentaose, the experimentally selected concentration of 0.4 μM fell within the predicted optimal range. Thus, the computational model was effective in identifying favorable operating conditions, although the experimentally selected concentrations did not necessarily coincide with the absolute predicted optimum. To reconcile the apparent discrepancy between the anisotropy-derived binding affinities and the PEGylated-ConA concentrations required to maximize the solvatochromic fluorescence response, we developed an expanded competitive binding model (see Section S1.7, Figure S8B (i and ii)). This model incorporates the experimentally determined anisotropy-derived dissociation constants together with glucose-ConA binding and includes an effective fluorescence-transducing site term to account for the fact that equilibrium complex formation and solvatochromic signal generation are not directly equivalent. Model fitting reproduced the experimentally observed PEGylated-ConA titration trends and indicated that the two anomers exhibit comparable equilibrium binding interactions with PEGylated-ConA, while differing in the effective fluorescence-transducing site-equivalent scaling factor contributing to the Cy5.5 fluorescence response. Accordingly, the higher PEGylated-ConA concentration required to maximize the β-mannopentaose solvatochromic signal is consistent with differences in fluorescence transduction efficiency rather than weaker equilibrium binding. Together, these results provide a quantitative framework linking the anisotropy-derived binding data and the solvatochromic fluorescence measurements.

### 3.6 Glucose Sensitivity with Cy5.5-Labeled Mannopentaoses Employing Fluorescence Anisotropy

To further elucidate the binding mechanism of the assay under varying glucose conditions, fluorescence anisotropy measurements were performed with different glucose concentrations, utilizing the competitive binding between PEGylated-ConA and Cy5.5-labeled mannopentaoses (Figure 5A). Fluorescence anisotropy measurements were performed as a mechanistic tool to probe glucose-dependent changes in PEGylated-ConA–mannopentaose complex formation and molecular mobility. It is important to note that anisotropy was not intended as a translational readout modality for in vivo sensing but rather to provide orthogonal biophysical evidence of competitive binding dynamics. The final sensing platform is designed for intensity-based NIR fluorescence readout using NIR LED excitation, which is compatible with nonpolarized optical detection through biological tissues. At low glucose concentrations, PEGylated-ConA forms large, slowly rotating complexes with the mannopentaose conjugates, producing high anisotropy. As glucose concentration increases, competitive displacement of the mannopentaose ligands occurs, resulting in smaller, faster-rotating species and a corresponding decrease in anisotropy. Thus, fluorescence anisotropy inversely correlates with glucose concentration, providing a direct readout of competitive binding dynamics. Notably, previous studies have established that glucose binding to ConA is reversible under physiological conditions.^38^ Both α- and β-Cy5.5-mannopentaose were tested across a broad glucose range (50–800 mg/dL) in TBS buffer to evaluate the influence of glycosidic linkage configuration. For Cy5.5-α-mannopentaose, anisotropy decreased smoothly from 0.25 at 50 mg/dL to 0.18 at 400 mg/dL before approaching saturation (Figure 5B), reflecting progressive glucose-mediated displacement while demonstrating robust sensitivity within the physiologically relevant range. The β-anomer exhibited a slightly higher initial anisotropy of 0.26 at 50 mg/dL, suggesting stronger initial binding consistent with the enhanced hydrogen bonding predicted in docking studies. Notably, β-mannopentaose exhibited a sharper and broader decrease in anisotropy (0.26 to 0.17) across 50–800 mg/dL glucose (Figure 5C), indicating enhanced dynamic displacement and extended sensing range. While α-mannopentaose saturated near 400 mg/dL, β-mannopentaose maintained responsiveness across the full tested glucose range, making it better suited for continuous monitoring and tight glycemic control applications. In addition, β-mannopentaose is approximately 10-fold less costly than the α-anomer, further supporting its selection as the preferred ligand for subsequent studies. Figure S8C shows that Cy5.5-β-mannopentaose maintained stable fluorescence over 30 days under the tested storage conditions. Within the resolution of this sampling scheme, no systematic change in fluorescence intensity was observed over time, indicating stable optical output of the labeled construct during storage.

**Figure 5:**
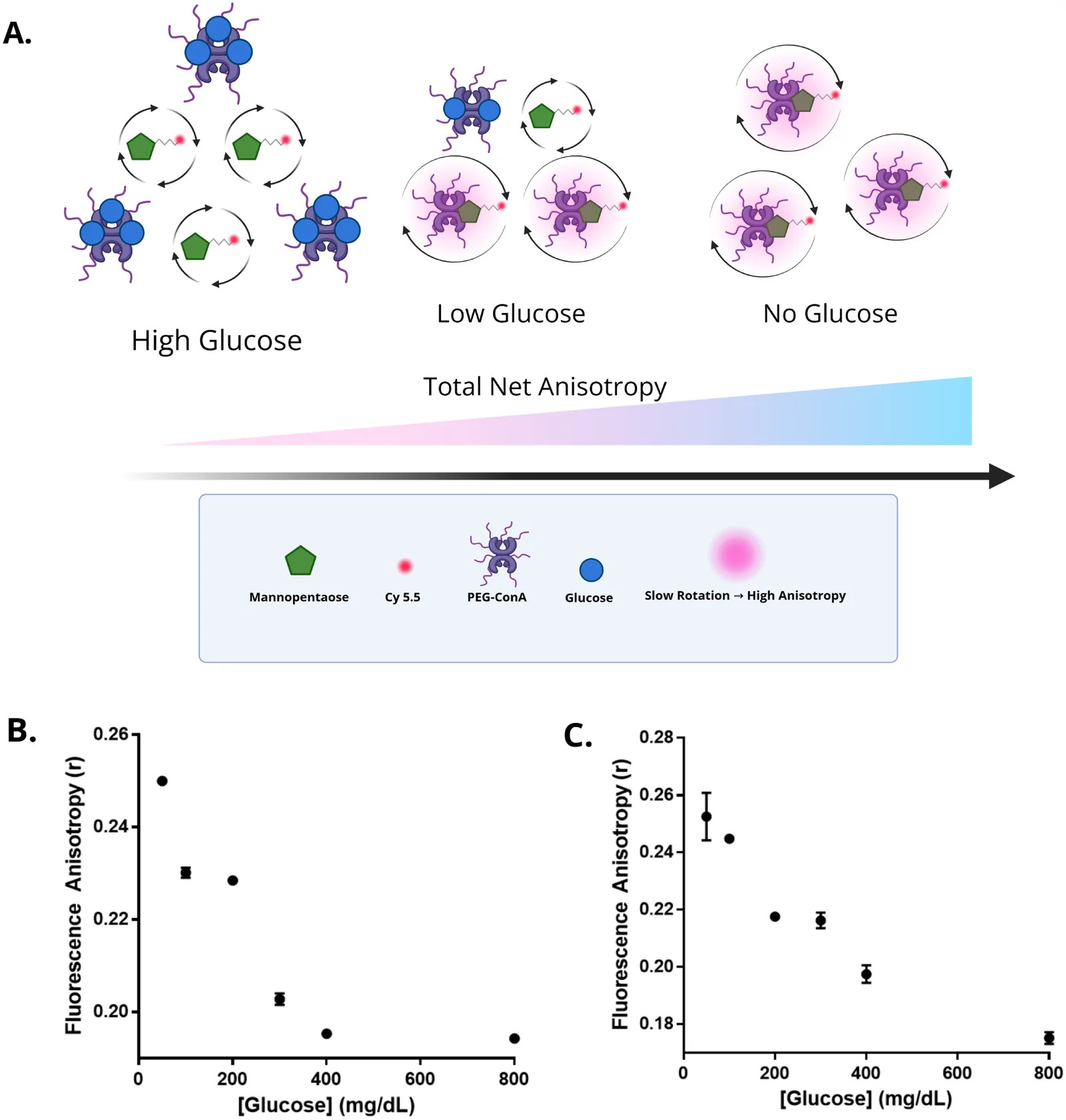
Fluorescence anisotropy-based glucose sensing of assays prepared from PEGylated-ConA and a Cy5.5-labeled mannopentaose. (A) Schematic representation of the competitive binding mechanism underlying the glucose-dependent fluorescence anisotropy. The net fluorescence anisotropy decreases proportionally with increasing glucose concentration. The inset legend indicates the molecular components involved in the assay, where slow rotational motion corresponds to a high anisotropy signal. (B) Fluorescence anisotropy profile of the assay with Cy5.5-α-mannopentaose demonstrated an inverse correlation between glucose concentration and anisotropy with a dynamic range spanning from 50 to 400 mg/dL. (C) Fluorescence anisotropy profile of the assay with Cy5.5-β-mannopentaose demonstrated a decreasing trend with slightly higher starting anisotropy values and a broader dynamic range (50–800 mg/dL), indicating consistency and reproducibility of the assay.

### 3.7 Validation of Glucose Sensing in Artificial ISF

To evaluate the translational potential of the PEGylated-ConA coupled β-mannopentaose–Cy5.5 sensing platform, we next investigated its performance in artificial ISF. The use of artificial ISF has been used widely before for glucose sensing studies.^50,51^ While our previous TBS buffer studies demonstrated that the system could detect glucose across a broad range (50–800 mg/dL) via competitive binding dynamics and fluorescence anisotropy, they do not account for the ionic strength, protein content, or potential interfering species present in interstitial fluid. Subcutaneous glucose concentrations are reported to be approximately 45–78% of blood glucose levels^52^ (Figure 6A). Based on this partitioning, hypoglycemic blood glucose (<70 mg/dL) corresponds to 32–55 mg/dL in ISF, normal blood glucose (100–140 mg/dL) corresponds to 50–110 mg/dL in ISF, and hyperglycemic blood glucose (200–400 mg/dL) corresponds to 90–250 mg/dL in ISF. For practical applicability, the sensing system must retain sensitivity across this physiologically relevant ISF range (30–250 mg/dL).

**Figure 6:**
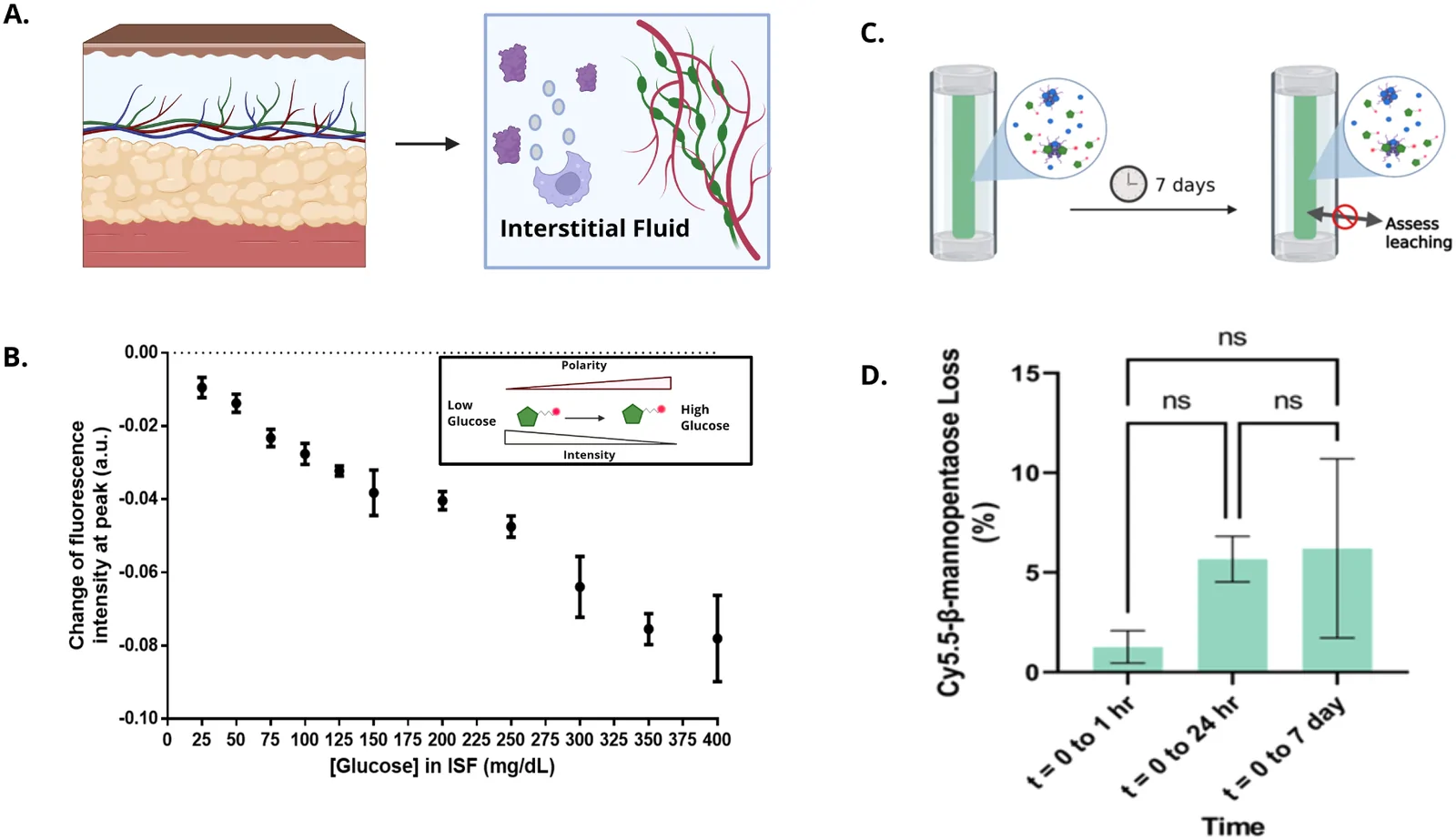
(A) Interstitial fluid in subcutaneous glucose monitoring applications. (B) Change in fluorescence intensity was recorded as a function of glucose concentration ranging from 25 to 400 mg/dL. (C) Schematic representation illustrating the retention of the glucose-sensing assay (e.g., PEGylated-ConA and Cy5.5-β-mannopentaose). (D) Loss of Cy5.5-β-mannopentaose at 1 h, 24 h, and 7 days. *p* < 0.05 vs prior time point; ^ns^ *p* > 0.05 vs prior time point.

To investigate the assay compatibility, we evaluated the assay across a wide range of glucose concentrations (Figure 6A), with particular emphasis on near-normal levels. Our experiments in ISF demonstrated reproducible solvatochromism (Figure 6B) across glucose concentrations of 25, 50, 75, 100, 125, 150, 200, 250, 300, and 400 mg/dL. Notably, the sensor maintained strong responsiveness in the 25–300 mg/dL range (corresponding to blood glucose 50–400 mg/dL), directly overlapping with the physiologically relevant subcutaneous window (hypo-, normal, hyperglycemic). The observed fluorescence response remained reproducible across the physiologically relevant glucose range. While the present study was designed to evaluate proof-of-concept sensing performance rather than analytical accuracy relative to a reference glucose analyzer, the assay exhibited consistent and monotonic signal modulation throughout the tested concentration range. The observed response indicates consistent signal modulation across the tested range, supporting reliable and monotonic assay behavior. At higher glucose concentrations (≥250 mg/dL in ISF, corresponding to ≥400 mg/dL blood glucose), solvatochromic responses remained monotonic, ensuring coverage across the full pathophysiological spectrum. To check the specificity, Cy5.5-coupled β-mannopentaose was incubated with varying glucose concentrations in ISF in the absence of PEGylated-ConA (Figure S9). No significant change in fluorescence intensity was observed across the tested glucose range (50–400 mg/dL), confirming that the observed decrease in fluorescence in the complete assay is specifically due to competitive displacement by glucose and not a direct effect on the fluorophore.

To further evaluate assay specificity in a physiologically relevant environment, interference studies were performed in artificial ISF containing a cocktail of fructose (100 μM), lactate (5 mM), ascorbic acid (200 μM), and uric acid (300 μM), representing physiologically relevant to mildly elevated extracellular concentrations (Figure S10). Glucose response curves were obtained in the presence of the interferent mixture across glucose concentrations ranging from 25 to 400 mg/dL. The resulting solvatochromic responses are similar to those obtained in artificial ISF without added interferents, which show the same trends with glucose-sensitivity solvatochromism trends. These results indicate that the fluorescence modulation remains predominantly governed by glucose-mediated competitive displacement of β-mannopentaose from PEGylated-ConA and is minimally affected by the tested interferents under the experimental conditions examined. These results indicate that the dynamic range of the β-mannopentaose–Cy5.5 conjugated PEGylated-ConA system in ISF (25–400 mg/dL) adequately encompasses the effective subcutaneous glucose range under hypo-, normal, and hyperglycemic conditions. The preservation of sensitivity in ISF further supports the mechanistic robustness of the assay, as specific PEGylated-ConA−β mannopentaose interactions dominate despite the presence of competing proteins, electrolytes, and metabolites.

### 3.8 Biosensor Proof-of-Concept: Incorporation of Assay into the Cavity of a Hydrogel Rod

Critical to the actualization of a cost-effective, injectable (e.g., to wrist subcutaneous tissue), and long-lasting biosensor is the successful confinement of the developed glucose-sensing assay. Thus, a proof-of-concept biosensor was constructed by housing the glucose-sensing assay into the cavity of a hydrogel rod (Figure 6C). As previously noted, this DN comb hydrogel was selected based on our prior reports that confirmed a small mesh size (1 nm < ξ < 3 nm) that permitted glucose diffusion (*D*_h_ ∼ 1 nm) but that would lead to the retention of assay molecules.^44^ Moreover, this membrane was mechanically robust and replicated the thermosensitivity profile to afford self-cleaning for reduced biofouling.^55^ Additionally, the use of nondegradable monomers with a carbon backbone can resist enzymatic degradation, maintaining structural integrity *in vivo*.^55^ 1.5 μL TRIS assay solution comprised of 0.4 μM PEGylated-ConA and 0.1 μM Cy5.5-β-mannopentaose was added to the cavity, and the ends were sealed with polycarbonate discs using an adhesive. The resulting biosensor’s small, cylindrical nature (∼2.5 mm × ∼11 mm, Ø × *l*) was intended to permit subcutaneous injection (Figure S11 and Video S1). Next, the biosensor was submerged in a highly concentrated solution of glucose (1000 mg/dL). By submerging the biosensor in a concentrated glucose solution, PEGylated-ConA and Cy5.5-β-mannopentaose remained unbound, allowing the potential for diffusion of Cy5.5-β-mannopentaose from the cavity to the surrounding solution. Based on the mesh size of the hydrogel, diffusion of PEGylated-ConA (*D*_h_ ∼ 30 nm)^38^ was not expected, and so was not evaluated.

Loss of Cy5.5-β-mannopentaose from the biosensor cavity was examined over a 7-day period (Figure 6D). Loss was negligible, with ∼6% lost after 7 days, and was not statistically greater than that observed after 1 or 24 h. These results point to excellent retention of the Cy5.5-β-mannopentaose, owing to the low mesh size of the hydrogel. The slight loss appears to occur within the first hour and may be due to the diffusion of loosely trapped or weakly associated Cy5.5-β-mannopentaose near the periphery of the hydrogel or in solvent-accessible regions. Such early-stage (≤24 h) diffusion is consistent with the establishment of a concentration gradient across the hydrogel–solution interface, driving initial outward diffusion.^56^ Overall, this represents a feasible strategy to house the glucose-sensing assay. To ensure continuous sensing is achieved, future work will explore signal stability in a simulated physiological environment.

## 4 Conclusion:

In this work, we report a nonenzymatic, near-infrared (NIR) fluorescence-based glucose sensing system based on PEGylated-ConA and Cy5.5−β-mannopentaose, integrated within a hydrogel-confined cylindrical construct. The design combines analytical sensitivity, structural stability, and a miniaturized form factor compatible with minimally invasive deployment, while maintaining functionality in physiologically relevant interstitial fluid environments. The central objective of this study was cost-performance optimization, where the β-mannopentaose-based system was systematically benchmarked against its α-anomer counterpart, which is more expensive and synthetically demanding. Molecular docking provided qualitative structural insights, indicating that both α- and β-mannopentaose can form stable interactions within the ConA carbohydrate recognition domain, with increased hydrogen-bonding contacts relative to shorter mannose oligosaccharides. These findings were consistent with experimental observations, where Cy5.5-labeled mannopentaoses were successfully synthesized and shown to form stable complexes with PEGylated-ConA. Fluorescence solvatochromism assays demonstrated glucose-responsive signal modulation arising from the competitive displacement of the mannopentaose ligand. Although the β-anomer required a higher receptor concentration to achieve saturation, it formed a stable and reproducible complex. Both α- and β-systems exhibited comparable glucose-dependent fluorescence responses across physiologically relevant ranges, with near-linear behavior between 25 and 300 mg/dL prior to saturation. Complementary fluorescence anisotropy measurements further supported the binding mechanism and revealed a broad dynamic response range (50–800 mg/dL), consistent with reversible competitive binding under glucose challenge.

The sensing performance was further validated in artificial interstitial fluid containing physiological salts and proteins, where reproducible responses were observed across hypoglycemic, normoglycemic, and hyperglycemic ranges (25–400 mg/dL), consistent with subcutaneous glucose distributions. While tissue phantoms or through-skin optical measurements were not performed in this study, the use of a NIR fluorophore (Cy5.5) provides an inherent advantage for reduced scattering and absorption in biological tissue, supporting its relevance for future transdermal optical interrogation. The observed inverse fluorescence response with increasing glucose concentration is an expected characteristic of the competitive binding mechanism and enables quantitative readout through appropriate calibration. Such displacement-based optical sensing strategies are well established and compatible with intensity-based detection formats. For device integration, the sensing chemistry was encapsulated within a low-mesh-size hydrogel (1 nm < ξ < 3 nm), forming a cylindrical construct (∼2.5 mm × ∼11 mm). The hydrogel effectively retained the Cy5.5-labeled ligand, with minimal leakage (∼6% over 7 days), while providing mechanical robustness and biocompatibility suitable for subcutaneous environments. However, it is important to note that the current work primarily demonstrates analyte retention and in vitro functional responsiveness. Future work will incorporate a dual-labeled FRET sensing architecture within the hydrogel construct and evaluate reversibility, glucose cycling behavior, signal drift, and long-term operational stability under physiologically relevant conditions. From a translational perspective, β-mannopentaose offers a clear economic advantage due to its lower cost and simpler synthetic accessibility, reducing variability associated with stereochemically complex synthesis. Although PEGylated-ConA is used at slightly different concentrations between systems, its contribution to overall assay cost (reagent-based only) is negligible at the working concentrations employed (Table S2). Similarly, Cy5.5 and buffer components contribute minimally to per-assay cost. Consequently, the primary cost and scalability advantage arises from ligand selection and synthetic feasibility.

Overall, this study establishes a cost-optimized, nonenzymatic NIR fluorescence glucose-sensing platform based on competitive binding interactions between PEGylated-ConA and mannopentaose ligands. The β-mannopentaose system demonstrates comparable sensing performance to the α-anomer while offering improved economic and practical advantages. The results further demonstrate stable operation in buffer and artificial interstitial fluid, along with effective confinement within a hydrogel matrix. While the system is currently validated in controlled environments, it provides a strong foundation for the development of minimally invasive optical glucose-sensing platforms, with future work directed toward achieving fully functional, continuous in vivo monitoring.

## Supplementary Material





## Notes

Injectability of Hydrogel (MP4)
Experimental methods; synthesis and characterization of Cy5.5-α-mannopentaose and Cy5.5-β-mannopentaose; mass spectrometry data; DLS analysis of PEGylated-ConA; molecular length analysis of mannopentaose ligands; nonspecific interaction and stability studies; binding solvatochromism model; specificity evaluation in artificial interstitial fluid; hydrogel injectability assessment; molecular docking scores; and cost comparison analysis (PDF)
