# Supplementary material for: β‑Anomeric Mannopentaose-Based Nonenzymatic NIR Glucose Sensor Enabling Cost-Effective Continuous Monitoring
Source: ACS Meas Sci Au. 2026 Jul 10;6(4):1179–93. doi: 10.1021/acsmeasuresciau.6c00076 (PMC13495729; doi:10.1021/acsmeasuresciau.6c00076)
Supplement: Supplementary file 1 [file tg6c00076_si_002.pdf]

β- Anomeric Mannopentaose-based Non-Enzymatic NIR Glucose Sensor enabling Cost-Effective Continuous Monitoring

Sayantan Tripathy* [1a,b], Diana Al Husseini[1 a,b], Carolina I. Martinez [b,c], Anthony Dongchau[a], Samyucktha Ganesapandian [a,b], Melissa A Grunlan [a,b,c], Gerard L Cote[a,b,d]

[a] Department of Biomedical Engineering, Texas A&M University, 600 Discovery Drive, College Station, TX, 77840-3006, USA

[b] Center for Remote Health Technologies and Systems, Texas A&M University, 600 Discovery Drive, College Station, TX, 77840-3006, USA

c Department of Chemistry, Texas A&M University, 580 Ross Street, College Station, TX, 77843, USA

[d] Department of Electrical and Computer Engineering, Texas A&M University, College Station, TX, 77840, USA

Corresponding author: s.tripathy@tamu.edu

**Contents**	**Page No**
**Experimental Section**	**2**
**Figure S1: Synthesis of Cy5.5-α-mannopentaose**	**12**
**Figure S2: Mass Spec Data of alpha mannopentaose**	**13**
**Figure S3: Mass Spec data of beta mannopentaose**	**14**
**Figure S4: DLS data of PEG ConA**	**15**
**Figure S5: Molecular Length of alpha mannopentaose**	**16**
**Figure S6: Molecular Length of beta mannopentaose**	**17**
**Figure S7: Optimization of the assay**	**18**
**Figure S8: Modeling and Stability assay of mannose ligand**	**19**

**Figure S9: Specificity determination of assay in ISF**	**20**
**Figure S10: Interference determination**	**21**
**Figure S11: Injectability of Hydrogel**	**22**
**Table S1: Docking Score**	**23**
**Table S2: Cost comparison**	**24**

1. Experimental Section:

1.1 Synthesis of Cy5.5-α-mannopentaose and Cy5.5-β-mannopentaose

To synthesize Cy5.5 conjugates, both α- and β-mannopentaose were individually functionalized using a bifunctional amine linker based on a previously reported method (Colvin et al., 2024). In each reaction, the mannopentaose (1 equiv.) was reacted with 1-(*N*-Boc-aminomethyl)-4-(aminomethyl)benzene (10 equiv.) and sodium cyanoborohydride ($NaBH_3CN$, 10 equiv.) in a DMSO/acetic acid (7:3, v/v) mixture. The reaction mixture was incubated at 36 °C for 5 days in an oil bath. Upon completion, the reaction mixtures were diluted with water to reduce the DMSO concentration below 1%, then lyophilized (LABCONCO FreeZone benchtop freeze-dryer). The resulting crude products were redissolved in water and purified by HPLC (Shimadzu Shim-pack GIS C18, 20 × 250 mm, 10 µm) using a 15-min linear gradient from 0% to 60% acetonitrile (solvent B, with 0.1% TFA) in water (solvent A, with 0.1% TFA) at a flow rate of 10 mL/min. Elution was monitored at 220 nm, and product identity was confirmed by electrospray ionization mass spectrometry (+ESI) using a Thermo Scientific Q Exactive Focus via direct infusion. The purified conjugates were then deprotected by treatment with a cleavage solution of trifluoroacetic acid (TFA), triisopropylsilane (TIPS), and water (95:2.5:2.5, v/v/v) for 2 hr at room temperature (RT). Deprotected products were precipitated by the addition of cold diethyl ether (−20 °C) and collected by centrifugation (4000 rpm, 15 min, 4 °C). The resulting solids were washed with water and lyophilized. For fluorophore labeling, the free amine on the linker was reacted with the Cy5.5 NHS ester (2 equiv.) in the presence of DIPEA (4 equiv.) in DMF. The reaction was shaken at RT for 24 hr, yielding the Cy5.5 conjugates.

1.2 Characterization of Cy5.5-α-mannopentaose and Cy5.5-β-mannopentaose

The Cy5.5-α-mannopentaose and Cy5.5-β-mannopentaose products were purified via HPLC (as above) with a linear gradient of 5% to 95% solvent B over 25 min. Detection was performed at

600 nm to monitor Cy5.5. Product identity was confirmed by (+) ESI mass spectrometry. The concentration of each final product was quantified by UV–vis absorbance at ~680 nm using a Cary 300 spectrophotometer, based on the extinction coefficient of Cy5.5 NHS ester (ε = 198,000 M^{-1} cm^{-1}).

1.3 Synthesis and characterization of PEGylated-ConA

Primary amines on ConA were PEGylated using a 5 kDa mPEG propionic acid NHS ester (mPEG-NHS). ConA (10 mg/mL) was first dissolved in a carbonate–bicarbonate buffer, and its binding sites were protected by the addition of methyl-α-D-mannopyranoside (MaM, 1.9 mg/mL). The mixture was gently mixed for 20 min to ensure binding. PEGylation was initiated by adding mPEG-NHS at a 32:1 molar ratio relative to ConA, and the reaction proceeded for 4 hr on a rocking platform at RT, followed by an extended incubation at RT for at least 24 hr. Purification was carried out using 2 mL Amicon Ultra-2 centrifugal filters (30 kDa MWCO) with seven washes of either water or TBS (pH 7.4), spun at 3000g for 15 min per wash at 4 °C. The protein was stored with TBS (pH 7.4). Protein concentration was determined by UV–Vis absorbance at 280 nm (ε = 118,560 M^{-1} cm^{-1}) using a Cary 300 spectrophotometer. Aggregation and size distribution were evaluated via dynamic light scattering (DLS) using a Zetasizer Nano, reporting both Z-average and polydispersity index (PDI).

1.4 Molecular docking studies with chimera

The 3D crystal structure of Concanavalin A (ConA) was obtained from the Protein Data Bank (PDB ID: 1JBC). The 3D structures of α- and β-mannopentaose were generated in UCSF Chimera using their corresponding Simplified Molecular Input Line Entry System (SMILES) strings retrieved from PubChem. Prior to docking, both receptor and ligand structures were prepared using standard preprocessing steps. All non-standard residues, including metal ions and cofactors, were removed from the receptor structure. Due to computational constraints, full energy minimization was not performed. Structure preparation was carried out using the DockPrep module in UCSF Chimera, which included removal of water molecules, correction of truncated side chains, addition of hydrogen atoms, assignment of partial charges, and conversion of structures to Mol2 format. Molecular docking simulations were carried out using AutoDock Vina to predict ligand binding poses and calculate binding affinities. Grid box parameters were defined as follows: center_x = 49, center_y = 55, center_z = 32 with dimensions of size_x = 40, size_y = 40, size_z = 40. Across all docking runs, the exhaustiveness was set to 8, the number of binding modes to generate was 9, and the energy range was defined as 3. Default parameters were used for any remaining settings. All other parameters were kept at default values. The resulting docking poses were visualized using ChimeraX. Hydrogen bonds and steric clashes were identified using the H-bonds and clashes commands, respectively. Hydrogen bonds were detected based on atom type and geometric criteria, while close contacts indicating unfavorable steric interactions were also highlighted.

1.5 Fluorescence anisotropy binding studies

Equilibrium binding experiments were conducted using a QuantaMaster spectrofluorometer (PTI) by recording the fluorescence emission spectra of Cy5.5-α-mannopentaose and Cy5.5-β-mannopentaose. The measurements were performed using polarizers placed in the excitation and emission path of the light wave . The polarizers were set in either vertical (V) or horizontal (H) orientations, where the vertical portion is defined as perpendicular to the plane of the excitation beam, and the horizontal direction is perpendicular to the vertical. I_{VH} represents the fluorescence intensity measured with a vertical excitation polarizer and a horizontal emission polarizer, I_{VV} represents the fluorescence intensity with both excitation and emission polarizers set vertically, and G denotes the correction factor of the instrument ($G = I_{VH}/I_{VV}$). Fluorescence anisotropy (r) was calculated using equation below

$$r = \frac{I_{VV} - GI_{VH}}{I_{VV} + 2GI_{VH}} (1)$$

All measurements were performed in TRIS buffer (pH 7.4) using an excitation wavelength of 680 nm. Each cuvette contained a fixed concentration (0.1 µM) of Cy5.5-labeled mannopentaose, to which serial dilutions of ConA or PEGylated-ConA were added. Sufficient time (around 5 minutes) was allowed for the system to reach equilibrium.

1.6 Glucose assay optimal concentration studies

The concentration of PEGylated-ConA was optimized through a fluorescence intensity study. Fluorescence changes were normalized using a reference-based approach, defined as $(F - F_{ref})/F_{ref}$, where F_{ref} represents the average fluorescence intensity of the reference binding state. This normalization allows comparison of relative signal deviations across different PEGylated-ConA concentrations. Serial dilutions of PEGylated-ConA were prepared in TBS buffer (pH = 7.4), with each dilution made up to a total volume of 2 mL in 2 mL centrifuge tubes. A 0.1 µM concentration of Cy5.5-α-mannopentaose or Cy5.5-β-mannopentaose was added to each dilution. To evaluate the effect of glucose, 10 µL of TBS was added to three wells as no-glucose controls, while 10 µL of a 12,064 mg/dL glucose stock solution was added to another three wells containing the assay, in the treated samples. The well plate was then placed into a TECAN Infinite 200 Pro M Nano plate reader, and fluorescence intensity was measured. Excitation was set at 655 nm, and emission spectra were collected from 690 nm to 720 nm. Differences in binding between PEGylated-ConA and Cy5.5-α-mannopentaose or Cy5.5-β-mannopentaose, in the presence and absence of glucose, resulted in varying fluorescence intensity differences across the PEGylated-ConA concentration series. The PEGylated-ConA concentration that produced the greatest fluorescence intensity

difference between the no-glucose and glucose-treated samples was identified as the optimal concentration for subsequent assays.

1.7 Modeling solvatochromism: α-mannopentaose versus β-mannopentaose

Because fluorescence anisotropy reports the formation of a rotationally restricted PEGylated-ConA–mannopentaose complex, whereas the solvatochromic fluorescence response depends on both complex formation and whether Cy5.5 is placed into a fluorescence-modulating microenvironment, a model was created to reconcile the experimental results from the anisotropy and solvatochromism experiments. Therefore, the PEGylated-ConA concentration required for maximal solvatochromic response should not be interpreted as a direct proxy for equilibrium binding affinity.

For each anomer i, where $i = \alpha$or β, mannopentaose and glucose was modeled as competing for an effective PEGylated-ConA site pool:

$$C_i^* + M_i \rightleftharpoons CM_i^* \quad (1)$$

$$C_i^* + G \rightleftharpoons CG_i^* \quad (2)$$

where $C_i^*$is the free effective PEGylated-ConA site concentration relevant to Cy5.5 fluorescence transduction, $M_i$is free Cy5.5-labeled mannopentaose, $G$is free glucose, $CM_i^*$is the fluorescence-transduction-relevant PEGylated-ConA–mannopentaose complex, and $CG_i^*$is the glucose-bound effective PEGylated-ConA state.

The corresponding dissociation constants are:

$$K_{d,M,i} = \frac{[C_i^*][M_i]}{[CM_i^*]} \quad (3)$$

$$K_{d,G} = \frac{[C_i^*][G]}{[CG_i^*]} \quad (4)$$

The anomer-specific mannopentaose dissociation constants were fixed from the anisotropy measurements:

$$K_{d,M,\alpha} = 0.10\ \mu\text{M}$$
$$K_{d,M,\beta} = 0.07\ \mu\text{M}$$

The glucose–ConA dissociation constant was set to:

$$K_{d,G} = 2500\ \mu\text{M}$$

corresponding to 2.5×10^{-3} M.

To consider that not all PEGylated-ConA contribute equally to Cy5.5 solvatochromic modulation, an effective fluorescence-transducing site concentration was introduced:

$$[C]_{\mathrm{eff},i} = s_i[C]_0 \quad (5)$$

where $[C]_0$is the nominal added PEGylated-ConA concentration and $s_i$is an empirical effective-site scaling factor for anomer i. This scaling factor maps the nominal PEGylated-ConA concentration to the effective site concentration that participates in the fluorescence-transducing model. Because s_i can reflect binding-site valency, PEGylated-ConA heterogeneity, site accessibility, and anomer-dependent Cy5.5 orientation, it is not interpreted as a simple physical fraction but as an effective fluorescence-transducing site-equivalent scaling factor.

The effective site mass balance is:

$$[C]_{\mathrm{eff},i} = [C_i^*] + [CM_i^*] + [CG_i^*]$$

or:

$$s_i[C]_0 = [C_i^*] + [CM_i^*] + [CG_i^*] \quad (6)$$

The mannopentaose and glucose mass balances are:

$$[M]_0 = [M_i] + [CM_i^*] \quad (7)$$

$$[G]_0 = [G] + [CG_i^*] \quad (8)$$

Combining the binding equilibria with these mass balances gives:

$$[CM_i^*] = \frac{[C_i^*][M]_0}{K_{d,M,i}+[C_i^*]} \quad (9)$$

and:

$$[CG_i^*] = \frac{[C_i^*][G]_0}{K_{d,G}+[C_i^*]} \quad (10)$$

Substitution into the effective PEGylated-ConA site balance gives the competitive binding equation:

$$s_i[C]_0 = [C_i^*] + \frac{[C_i^*][M]_0}{K_{d,M,i}+[C_i^*]} + \frac{[C_i^*][G]_0}{K_{d,G}+[C_i^*]} \quad (11)$$

This equation was solved for $[C_i^*]$under each glucose condition. In the absence of glucose, the bound mannopentaose complex is denoted $[CM_i^*](0)$. Under glucose challenge, the bound mannopentaose complex is denoted $[CM_i^*](G)$. The glucose-induced mannopentaose displacement is then:

$$D_i = [CM_i^*](0) - [CM_i^*](G) \quad (12)$$

The measured solvatochromic fluorescence response was modeled as:

$$\Delta F_i = B_i + A_i D_i$$

where $B_i$is a baseline offset and $A_i$is the optical transduction scale converting glucose-induced displacement into fractional fluorescence change. After substitution, the fitted response equation becomes:

$$\Delta F_i = B_i + A_i\left(\frac{C_{0,i}^*[M]_0}{K_{d,M,i}+C_{0,i}^*} - \frac{C_{G,i}^*[M]_0}{K_{d,M,i}+C_{G,i}^*}\right) \quad (13)$$

where $C_{0,i}^*$ is the free effective PEGylated-ConA site concentration in the absence of glucose and $C_{G,i}^*$is the free effective PEGylated-ConA site concentration after glucose challenge.

This formulation explicitly incorporates the different anisotropy-derived binding affinities through $K_{d,M,i}$, while allowing the solvatochromic response to depend on the effective PEGylated-ConA site pool that generates a Cy5.5-responsive bound state.

The best-supported model by corrected Akaike information criterion was the competitive displacement model with a shared optical transduction scale and anomer-specific effective-site scaling factors:

$$AICc = -311.79$$

This $AICc$ score was the lowest for when s_i is distinct for both anomers while A_i was kept equivalent for both anomers out of all 4 model variants tested: both terms distinct ($AICc$ = -308.81), both terms equivalent ($AICc$ = -307.53), and A_i distinct while s_i was shared ($AICc$ = -304.60). Thus, s_i distinct for both anomers while A_i was kept equivalent for both anomers was the model chosen, shown in Figure S8Bi and S8Bii.

The fitted parameters were:

$$s_\alpha = 1.1008$$

$$s_\beta = 0.7470$$

$$\frac{s_\beta}{s_\alpha} = 0.679$$

$$A_\alpha = A_\beta = 6.8056$$

$$B_\alpha = 0.0632$$

$$B_\beta = 0.0431$$

Goodness-of-fit analysis showed that the model captured the major concentration-dependent trends in the PEGylated-ConA titration data:

$$RMSE = 0.021$$

Because the fluorescence response was expressed as fractional fluorescence change, the RMSE corresponds to an average prediction error of approximately 2.1 percentage points. The effective-site ratio remained below unity:

$$\frac{s_\beta}{s_\alpha} = 0.679$$

with a 95% confidence interval of:

$$95\%\ CI = [0.586, 0.782]$$

This result indicates that, at the same nominal PEGylated-ConA concentration, the β system has a smaller effective pool of fluorescence-transducing bound states than the α system. Therefore, the higher PEGylated-ConA concentration required for maximal β-mannopentaose solvatochromic response is quantitatively consistent with reduced effective fluorescence-transducing site availability rather than any suggested weaker equilibrium binding that would contradict the comparative binding between the two anomers found from the anisotropy experiment.

The anisotropy binding data were refit using a one-site binding model:

$$r(C) = r_{\min} + \frac{(r_{\max} - r_{\min})C}{K_a + C} \quad (14)$$

where $r(C)$is the measured fluorescence anisotropy at PEGylated-ConA concentration C, $r_{\min}$is the fitted lower anisotropy limit, $r_{\max}$is the fitted upper anisotropy limit, and $K_d$is the apparent dissociation constant. This model was used for both Cy5.5-α-mannopentaose and Cy5.5-β-mannopentaose.

For Cy5.5-α-mannopentaose, the refit gave:

$$K_{d,\alpha} = 0.1008\ \mu\text{M}$$

with a 95% confidence interval of:

$$95\%\ CI = [0.050, 0.200]\ \mu\text{M}$$

The fitted lower and upper anisotropy limits were:

$$r_{\min,\alpha} = 0.2966$$
$$r_{\max,\alpha} = 0.6799$$

The goodness-of-fit statistics were:

$$R^2 = 0.9414$$
$$R^2_{\text{adj}} = 0.9268$$
$$RMSE = 0.0336$$

For Cy5.5-β-mannopentaose, the refit, Figure 4 B and C, gave:

$$K_{d,\beta} = 0.0737\ \mu\text{M}$$

with a 95% confidence interval of:

$$95\%\ CI = [0.036, 0.156]\ \mu\text{M}$$

The fitted lower and upper anisotropy limits were:

$$r_{\min,\beta} = 0.1987$$
$$r_{\max,\beta} = 0.6518$$

The goodness-of-fit statistics were:

$$R^2 = 0.9294$$
$$R^2_{\text{adj}} = 0.9118$$
$$RMSE = 0.0448$$

These residual plots (Figure S8A) show that the largest deviations occur at intermediate PEGylated-ConA concentrations; however, there is not systematic deviation overall. To better characterize the fit in light of these intermediate-concentration deviations, adjusted R-squared

values are reported alongside R-squared and provide a more conservative description of curve quality.

Importantly, the confidence intervals for the two K_d values overlap:

$$K_{d,\alpha}: 0.050 - 0.200\ \mu\text{M}$$
$$K_{d,\beta}: 0.036 - 0.156\ \mu\text{M}$$

Therefore, β-mannopentaose has comparable anisotropy-derived affinities to α-mannopentaose, with β-mannopentaose showing a slightly lower fitted point estimate (Figure 4 B and C). Regarding propagation of the K_d uncertainty into the optimal-concentration predictions, the computational sensitivity plot served as an initial screening step to identify candidate concentration regimes, which were then experimentally optimized in the fluorescence study. The reported confidence intervals on K_d therefore bound the input uncertainty to this screening step, and because the final operating concentrations were established empirically rather than taken directly from the sensitivity model, the predicted optimal regimes are confirmed by the experimental data rather than relied upon as standalone predictions. The screening role of the sensitivity model thus ensures that K_d uncertainty affects only the screening model and was mitigated because the final operating concentrations were empirically optimized.

1.8 Preparation of artificial interstitial fluid (ISF)

The ISF was prepared by dissolving sodium chloride (Sigma, 6.3 gm/L), potassium chloride (Sigma, 0.26 gm/L), calcium chloride (Sigma, 0.17 gm/L), magnesium sulfate (Sigma, 0.17 gm/L), sodium bicarbonate (Sigma, 2.18 gm/L), monobasic sodium phosphate (Sigma, 0.26 gm/L), and sucrose (Sigma, 2.6 gm/L) in 1 L of deionized water [1,2]. The pH was adjusted to 7.4 by addition of hydrochloric acid, and 2% (w/v) bovine serum albumin (Sigma, BSA) was added to approximate the protein content of native interstitial fluid. A 0.1 µM solution of Cy5.5-β-mannopentaose was prepared in the ISF, followed by the addition of PEGylated-ConA at the previously optimized concentration. Serial additions of glucose were performed to achieve final concentrations of 50, 75, 100, 125, 150, 300, 400, and 800 mg/dL, spanning physiologically relevant normoglycemic and hyperglycemic ranges. For solvatochromism measurements, samples were transferred to a 96-well plate and analyzed using a TECAN Infinite 200 Pro M Nano plate reader. Excitation was set at 655 nm, and emission spectra were collected from 690–720 nm. Changes in peak fluorescence intensity were calculated relative to no-glucose controls. For fluorescence anisotropy measurements, the same assay mixture was used. Anisotropy was recorded with excitation at 680 nm and emission spectra at 695-740 nm using both polarizers. Anisotropy values were calculated using standard equation (Equation 1).

1.9 Construction of biosensors and assay retention in hydrogel

To prepare biosensors, the glucose-sensing assay solution was placed into the cavity of the DN comb hydrogel rod and the ends sealed. Biosensors were constructed in a dark room to avoid premature excitation of the glucose-sensing assay. A Kimwipe and syringe were used to remove excess water from the surface and central cavity, respectively, of the hydrogel rod (~ 2.5 mm x ~ 10 mm, *Ø* x *l*), respectively. The central cavity was filled with 1.5 µL TRIS assay solution comprised of 0.4 µM PEGylated-ConA and 0.1 µM Cy5.5-β-mannopentaose. A cyanoacrylate glue (Loctite 416) was used to adhere polycarbonate disc (0.25 mm x 2.5 mm, *t* x *d*) to each end of the hydrogel rod. A 10-min cure time was utilized to ensure adequate adhesion between the rod and 'caps', and a DI water-soaked Kimwipe was placed over the hydrogel rods to minimize dehydration. The resulting biosensor (~ 2.5 mm x ~ 11 mm, *Ø* x *l*) (n = 3 per timepoint) was rinsed with DI water, blotted with a Kimwipe, and placed into a microcentrifuge tube containing a 150 µL solution of glucose dissolved in TBS (pH-7.6) (1000 mg/dL) at RT. A 50 µL aliquot of the surrounding solution was removed at a designated time point (i.e., t = 0 to 1 hr, t = 0 to 24 hr, and t = 0 to 7 days), and transferred to a microplate. Fluorescence intensity measurements were recorded (Tecan Infinite 200 Pro M Plex plate reader, excitation wavelength (λ_{ex}) = 655 nm, emission wavelength (λ_{em}) = 690 - 720 nm) and the % glucose-sensing assay leached was determined using the following equation:

$$Glucose - sensing\ assay\ loss\ (\%) = \frac{Measured\ peak\ \ \lambda_{em}\ intensity}{Initial\ \lambda_{em}\ intensity} \times (13)$$

The initial intensity was determined by injecting 1.5 µL glucose-sensing assay solution directly into 150 µL solution of glucose dissolved in TBS (1000 mg/dL).

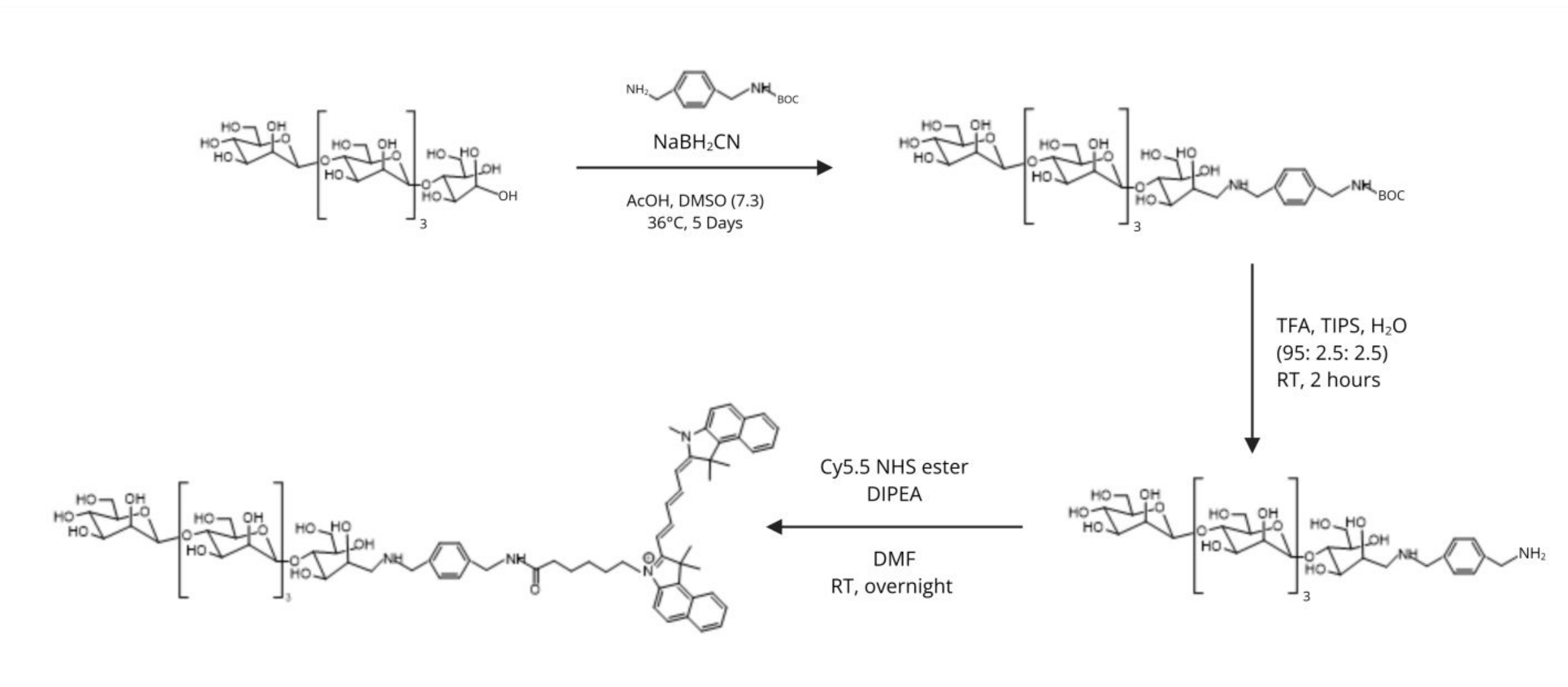

Figure S1: Synthesis of Cy5.5-α-mannopentaose. An analogous procedure was followed to synthesize Cy5.5-β-mannopentaose.

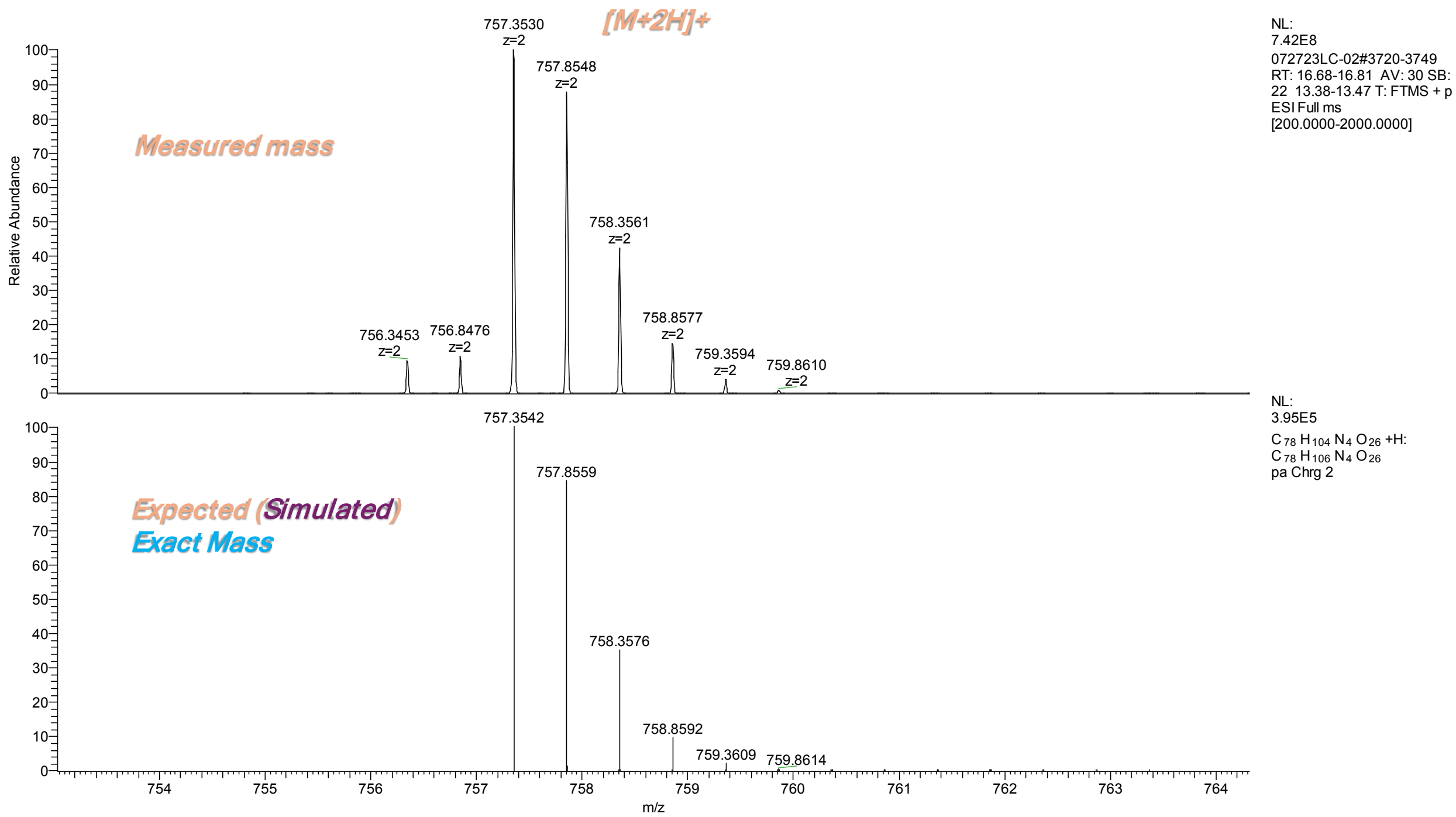

Figure S2: (+) ESI MS spectrum of α-mannopentaose showing the $[M{+}2H]{+}$ ion at m/z 757.3530 (z = 2). The measured isotopic pattern (756.34–759.86) matches the simulated exact masses, confirming the expected structure.

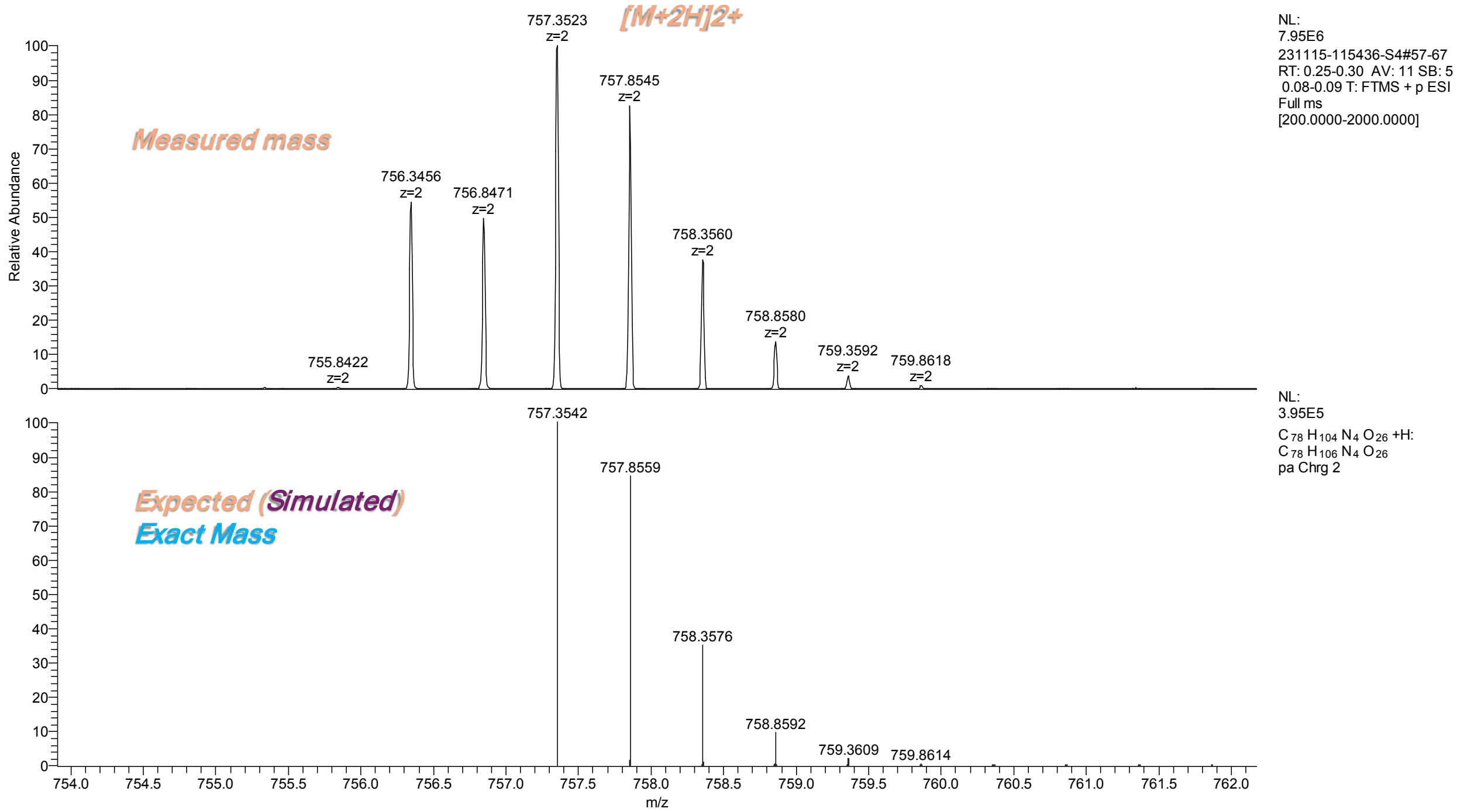

Figure S3: (+)ESI MS spectrum of β-mannopentaose showing the $[M{+}2H]2{+}$ion at m/z 757.3523 (z = 2). The measured isotopic distribution (755.8422–759.8618) closely aligns with simulated exact masses, confirming the expected molecular structure

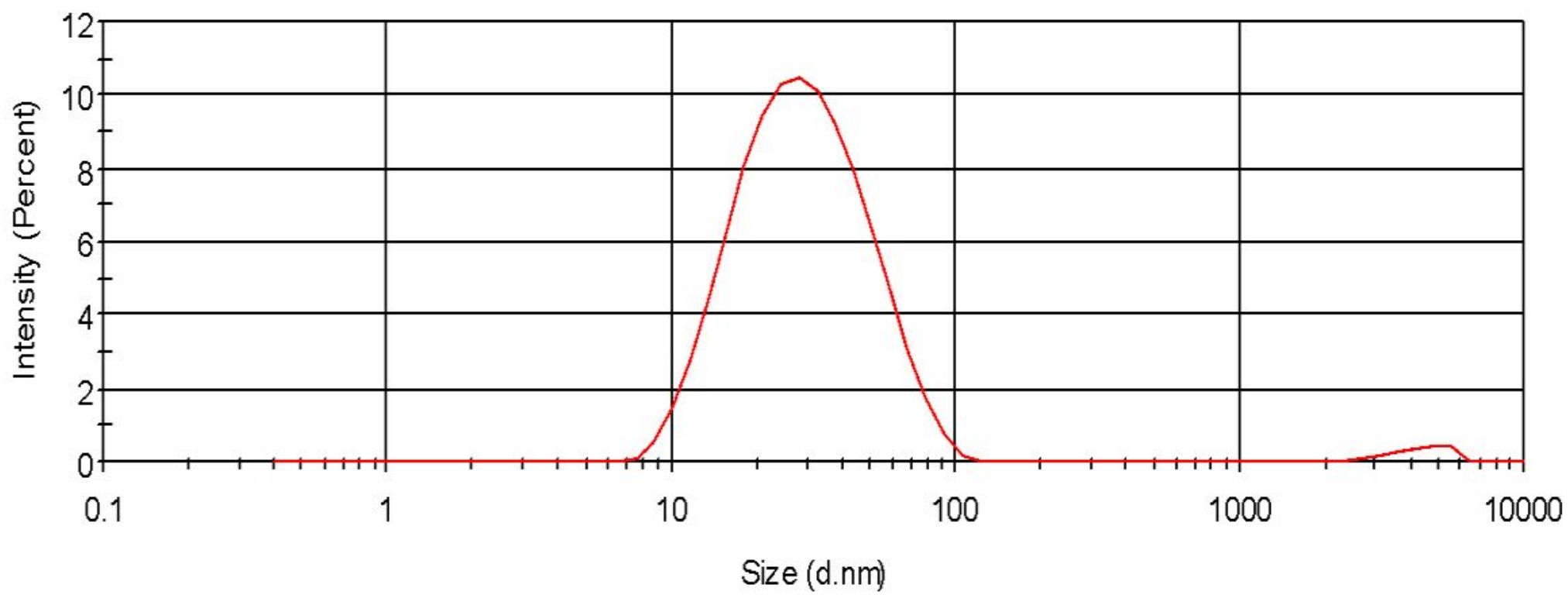

			Size (d.nm):	% Intensity:	St Dev (d.nm):
Z-Average (d.nm):	26.04	Peak 1:	32.30	98.2	16.76
PdI:	0.221	Peak 2:	4325	1.8	954.9
Intercept:	0.884	Peak 3:	0.000	0.0	0.000
Result quality :	Good				

Figure S4: DLS data of PEG ConA. The average size is 32 nm and PDI is 0.22, which confirms that there is less aggregation.

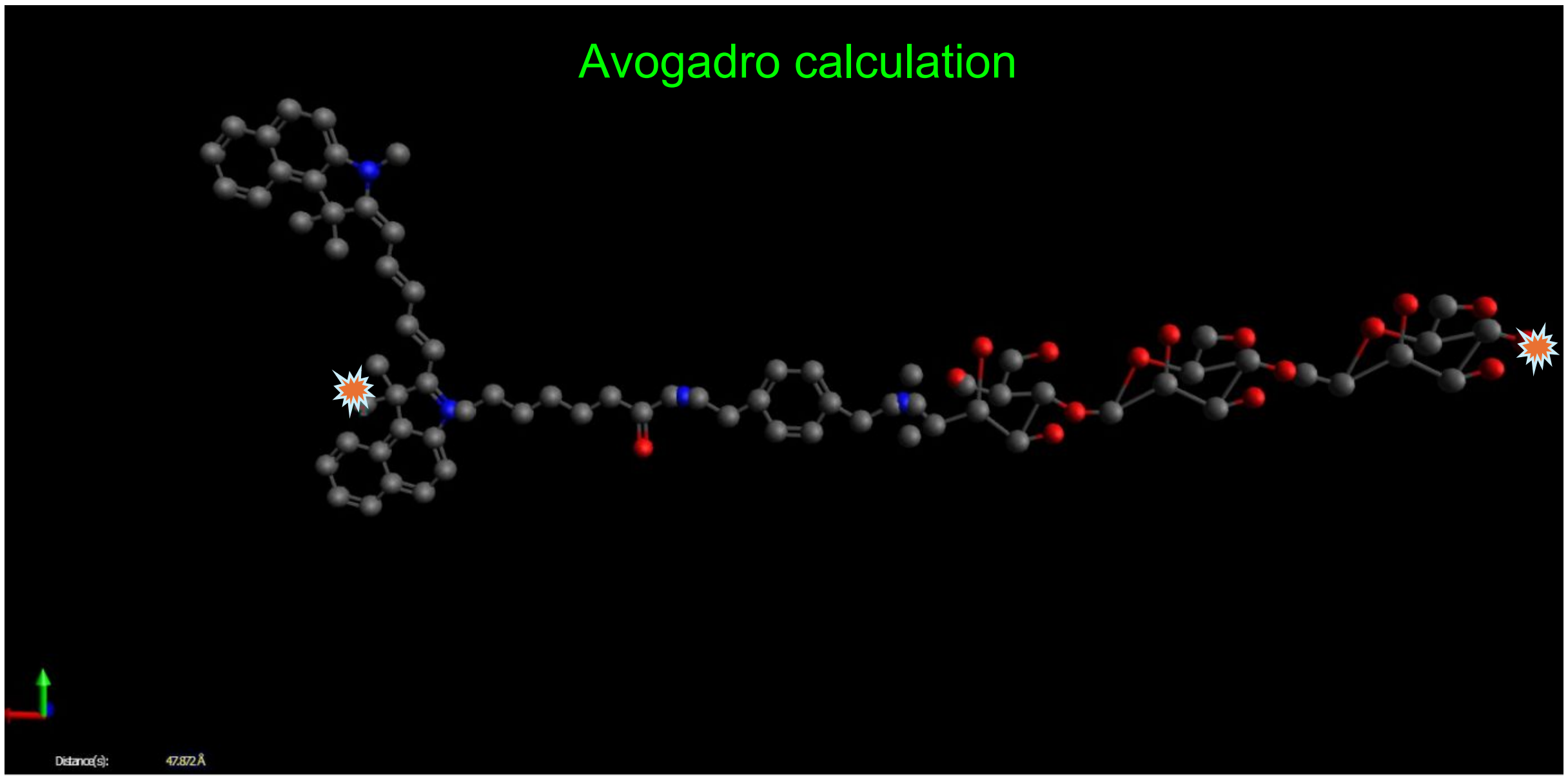

47.872 Å **mannopentaose**

Figure S5: Molecular length of α-mannopentaose

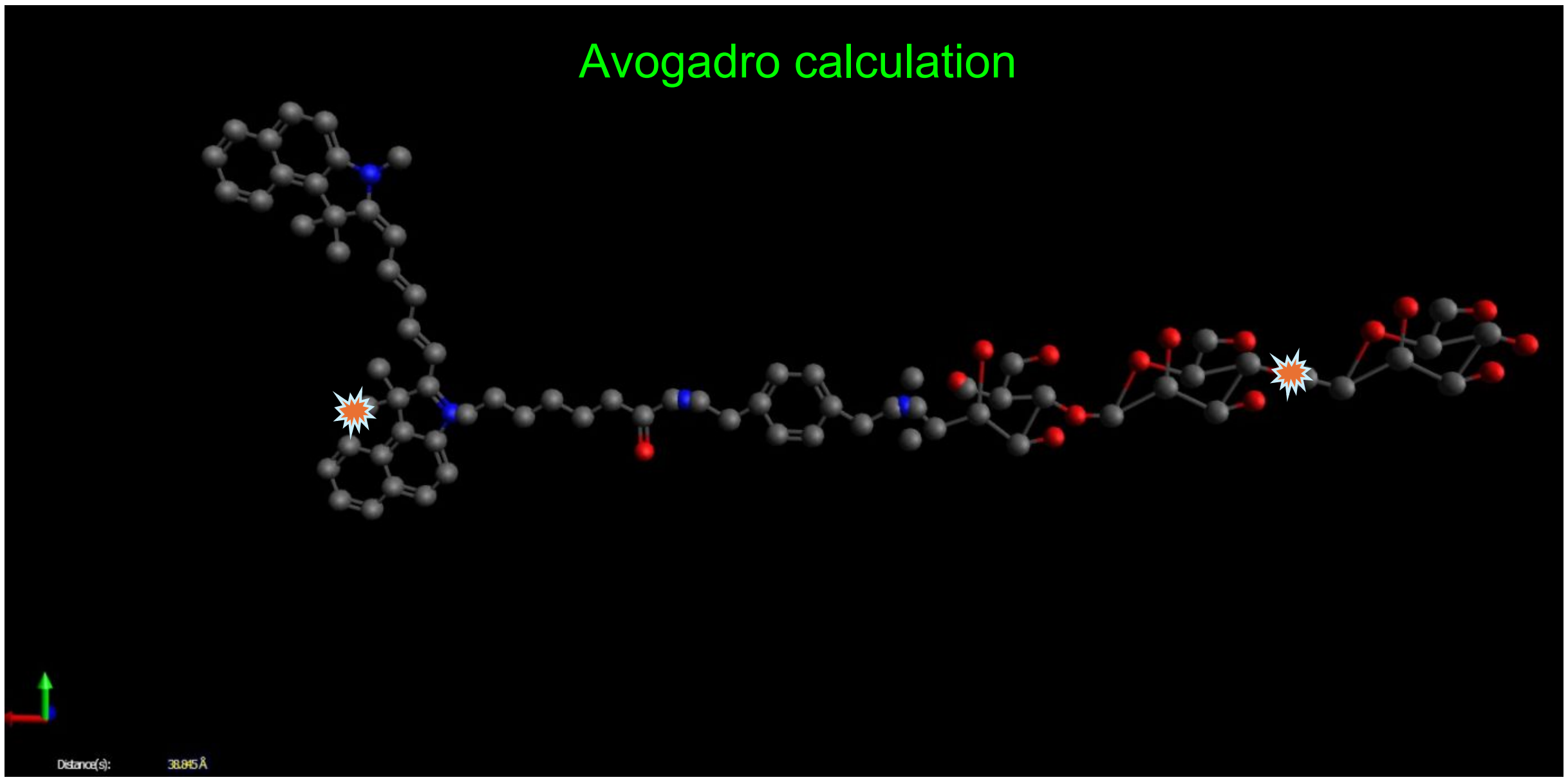

38.845 Å | **mannotetraose**

Figure S6: Molecular length of β-mannopentaose.

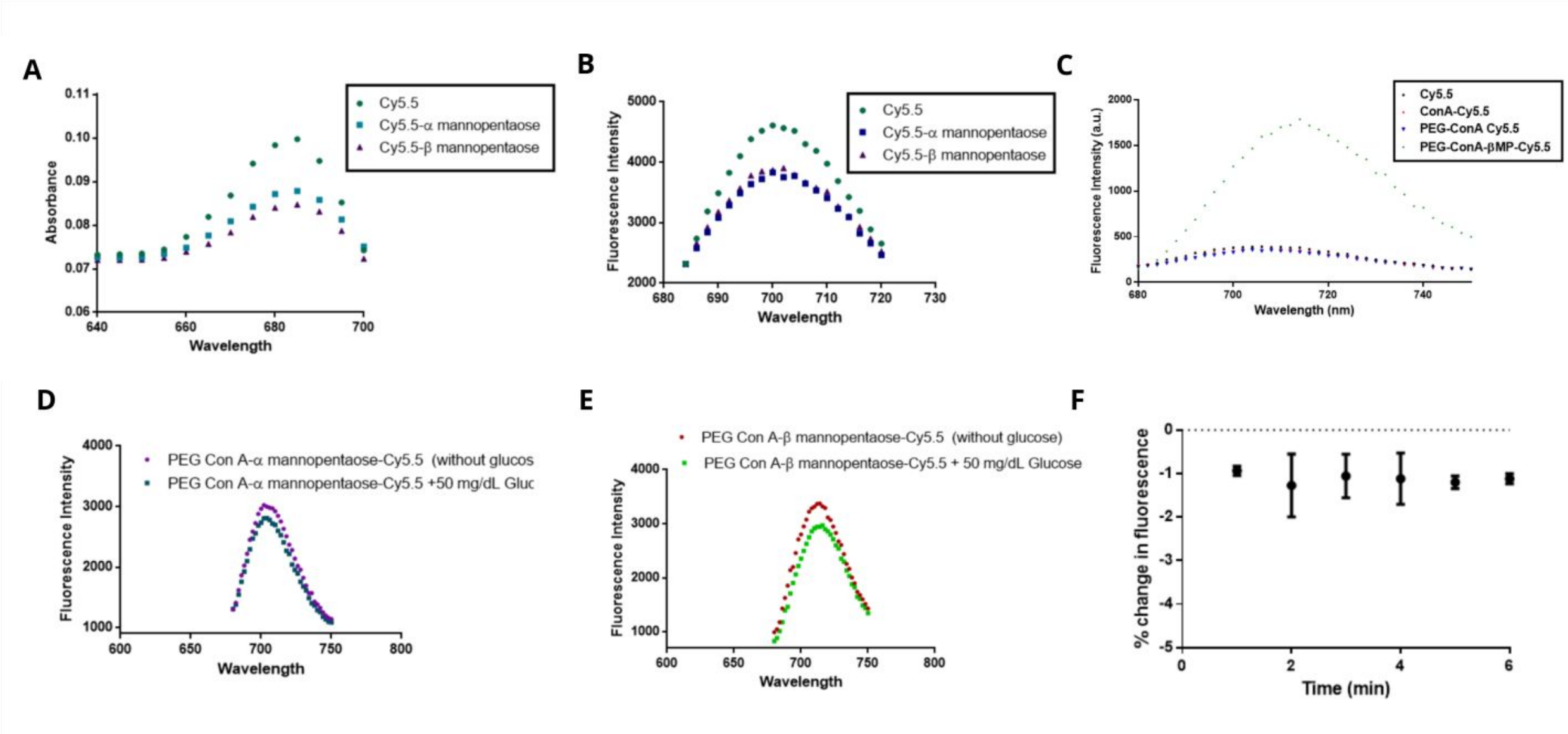

Figure S7: (A) UV–Vis absorbance of free Cy5.5, Cy5.5-α-mannopentaose, Cy5.5-β-mannopentaose demonstrating preservation of characteristic NIR optical properties following conjugation (B) Fluorescence spectra of free Cy5.5, Cy5.5-α-mannopentaose, Cy5.5-β-mannopentaose. **(C)** Fluorescence spectra of different Cy5.5-ConA formulations following centrifugal filtration (30 kDa). Samples included: free Cy5.5 in buffer (black), Cy5.5 incubated with ConA (red), Cy5.5 incubated with PEGylated-ConA (blue), and β-mannopentaose conjugatedCy5.5 incubated with PEGylated-ConA (green). (D) Representative fluorescence emission spectra of the Cy5.5–α-mannopentaose–PEGylated-ConA complex in the absence and presence of glucose (E) Representative fluorescence emission spectra of the Cy5.5–β-mannopentaose–PEGylated-ConA in the absence and presence of glucose. (F) Photobleaching experiment.

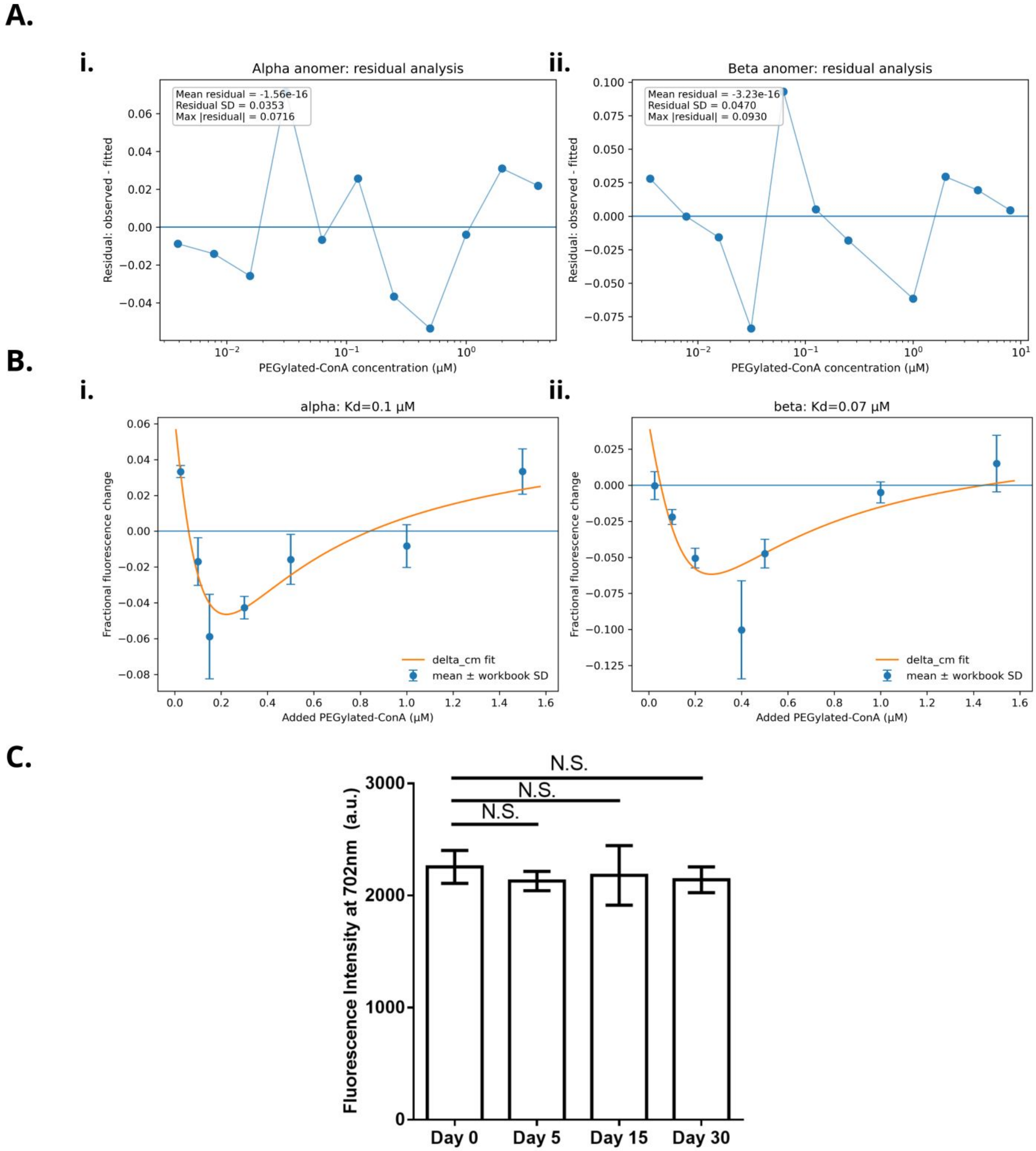

Figure S8: (A) Residuals of the binding isotherms for PEGylated-ConA with Cy5.5-α-mannopentaose (i) and Cy5.5-β-mannopentaose (ii). (B) Fitted model of solvatochromism transduction of α-mannopentaose (i) versus β-mannopentaose (ii). (C) Stability of Cy5.5 β-mannopentaose over 30 days.

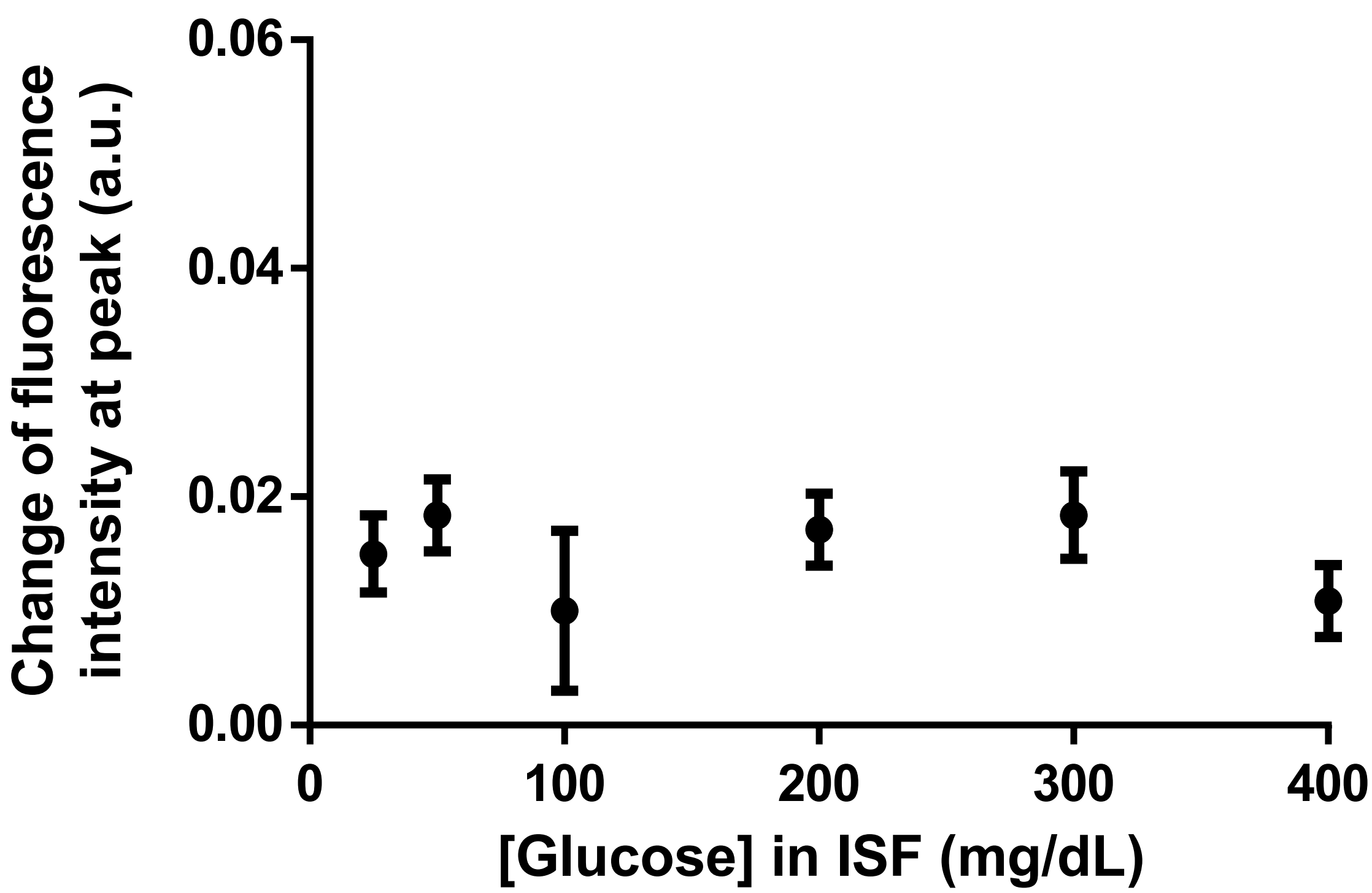

Figure S9: Change in fluorescence intensity of Cy5.5–β-mannopentaose upon addition of glucose in ISF. Fluorescence intensity at the emission peak was monitored as a function of glucose concentration (0–400 mg/dL). There is no decrease in fluorescence intensity was observed.

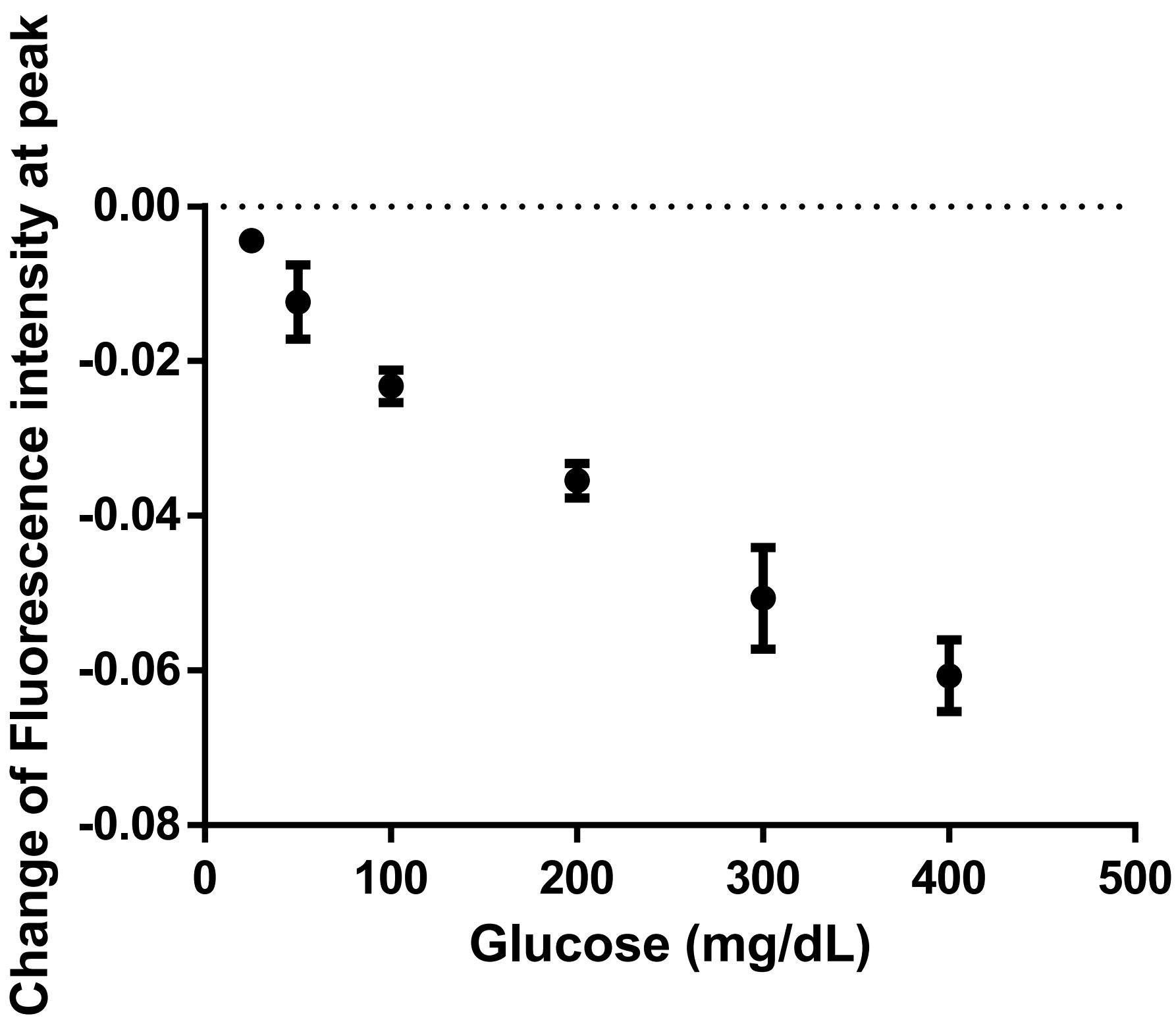

Figure S10: ISF Interference study

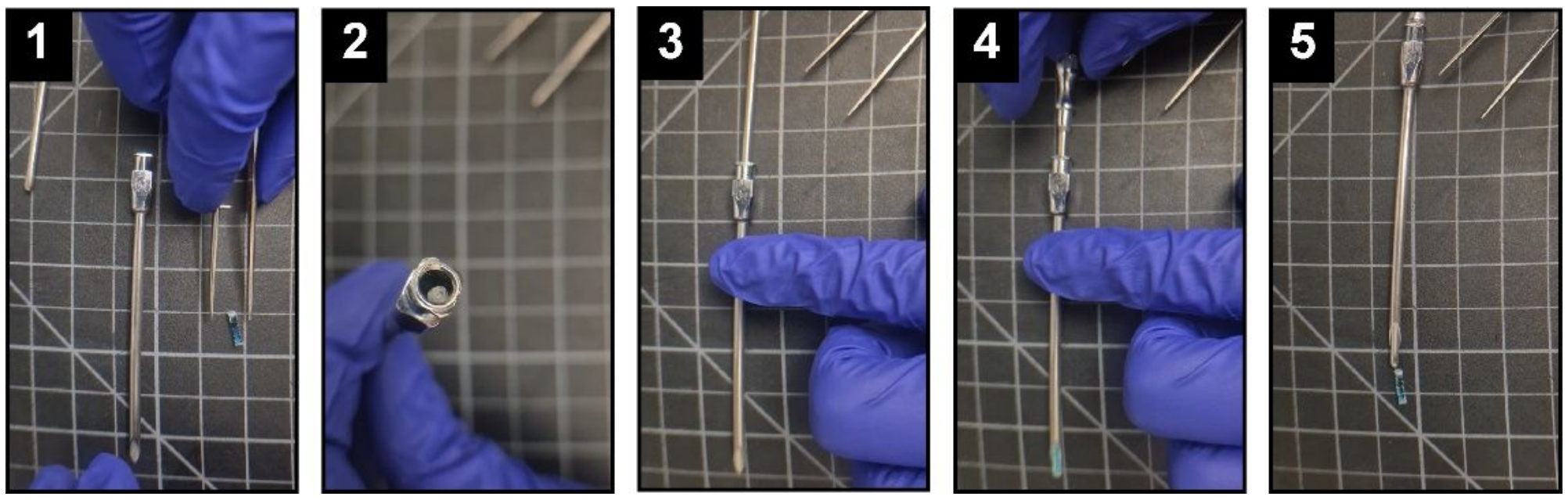

Figure S11: (**1**) A CAB was loaded with a blue dye and (**2**) subsequently placed within the inner diameter of a 10G trocar needle. (**3-5**) Once aligned, the CAB was successfully pushed through the trocar needle.

Table S1: Summary of molecular docking study

**Compound**	**Docking Score**	**Clashes**	**H bond**
α-mannopentaose	-5.4	0	5
β-mannopentaose	-5	2	9
Mannotetraose	-5.8	1	2
Mannotriose	-5.4	0	0
Mannobiose	-5.2	1	1

Table S2: Reagent based Cost comparison

**Reagents / Materials**	**Cost per assay (α-system)**	**Cost per assay (β-system)**
Mannopentaose	~$2.80	~$0.30
PEGylated-ConA	~$0.02	~$0.06
Cy5.5 labeling (0.1 μM)	~$0.25	~$0.25
Linker & conjugation reagents	~$0.40	~$0.40
Chemicals & buffers	~$0.30	~$0.30
**Total**	**~$3.77**	**~$1.31**

Reference:

[1] J.H. Han, C.R. Kim, C.H. Min, M.J. Kim, S.-N. Kim, H.B. Ji, S.B. Yoon, C. Lee, Y.B. Choy, Microneedles coated with composites of phenylboronic acid-containing polymer and carbon nanotubes for glucose measurements in interstitial fluids, Biosens. Bioelectron. 238 (2023) 115571. https://doi.org/10.1016/j.bios.2023.115571.

[2] S. Lin, X. Cheng, J. Zhu, B. Wang, D. Jelinek, Y. Zhao, T.-Y. Wu, A. Horrillo, J. Tan, J. Yeung, W. Yan, S. Forman, H.A. Coller, C. Milla, S. Emaminejad, Wearable microneedle-based electrochemical aptamer biosensing for precision dosing of drugs with narrow therapeutic windows, Sci. Adv. 8 (2022) eabq4539. https://doi.org/10.1126/sciadv.abq4539.
